# Different ways of evolving tool-using brains in teleosts and amniotes

**DOI:** 10.1038/s42003-023-05663-8

**Published:** 2024-01-12

**Authors:** Pierre Estienne, Matthieu Simion, Hanako Hagio, Naoyuki Yamamoto, Arnim Jenett, Kei Yamamoto

**Affiliations:** 1 Paris-Saclay Institute of Neuroscience (NeuroPSI), Université Paris-Saclay, CNRS UMR9197, Saclay, 91400 France; 2 TEFOR Paris-Saclay, CNRS UAR2010, Université Paris-Saclay, Saclay, 91400 France; 3 Laboratory of Fish Biology, Graduate School of Bioagricultural Sciences, Nagoya University, Nagoya, 464-8601 Japan; 4 Institute for Advanced Research, Nagoya University, Nagoya, 464-8601 Japan; 5 Present Address: Université Paris-Saclay, UVSQ, EnvA, INRAE, BREED, Jouy-en-Josas, 78350 France

**Keywords:** Neuroscience, Evolution

## Abstract

In mammals and birds, tool-using species are characterized by their relatively large telencephalon containing a higher proportion of total brain neurons compared to other species. Some teleost species in the wrasse family have evolved tool-using abilities. In this study, we compared the brains of tool-using wrasses with various teleost species. We show that in the tool-using wrasses, the telencephalon and the ventral part of the forebrain and midbrain are significantly enlarged compared to other teleost species but do not contain a larger proportion of cells. Instead, this size difference is due to large fiber tracts connecting the dorsal part of the telencephalon (pallium) to the inferior lobe, a ventral mesencephalic structure absent in amniotes. The high degree of connectivity between these structures in tool-using wrasses suggests that the inferior lobe could contribute to higher-order cognitive functions. We conclude that the evolution of non-telencephalic structures might have been key in the emergence of these cognitive functions in teleosts.

## Abstract (web summary)

A neuroanatomical study of the brains of tool-using teleost fishes highlights the pallium and inferior lobe as the putative structures responsible for this behavior, and suggests that there are multiple ways of evolving tool-using brains.

## Introduction

In primates, the cerebral cortex is the center for higher-order cognition such as logical thinking or self-recognition. However, some birds, such as corvids and parrots, demonstrate comparable cognitive functions, even though they do not possess this six-layered cortical structure^1,2^. Remarkable behaviors indicative of so-called higher-order cognition include tool use^3,4^, which requires abilities of causal reasoning, planning, as well as fine object manipulation.

Encephalization, the increased relative mass of the brain compared to body mass^5^, has long been used as a proxy for intelligence in vertebrates, with highly encephalized species considered more intelligent^6,7^. Despite the high degree of encephalization in corvids and parrots, cognitive abilities in birds have long been underestimated due to their rather small brains compared to mammals. A more recent cell counting study has in fact revealed that the brains of parrots and songbirds are extremely neuron-dense and contain on average twice as many neurons as primate brains of the same mass^8^. Thus, some species of corvids and parrots have as many neurons in their pallium (the dorsal telencephalon that contains the cerebral cortex in mammals) as some species of primates^8^. This strongly suggests that mammals and birds have followed two independent trajectories of encephalization: an increase in cortical surface in mammals (with the cortex reaching a very large size in humans), and an increase in the neuronal density of the pallium in birds. Both trajectories led to an increase in the absolute number of telencephalic neurons in highly encephalized species of mammals and birds compared to poorly encephalized ones. In other words, encephalization in amniotes (the clade containing mammals and birds) is mostly a process of “telencephalization“^8–10^.

Teleost brains are generally much less encephalized compared to amniotes^5^. Nonetheless, some teleost fishes belonging to the family of wrasses (*Labridae*) exhibit tool use-like behavior^11^. Due to the lack of grasping appendages in teleosts, the only way of using a “tool” is holding it in their mouth. In most cases of tool use by wrasses, the fish grasps a shellfish (bivalves) in its jaws, takes it to a feeding station that is equipped with appropriate “anvils” embedded in the substrate (corals or rocks), and then crushes it open by repeated slamming against the anvil^12–14^. Such behavior does not appear to be a genetically programmed “fixed action pattern”, since it can be observed also in captivity, in a slightly modified form. For example, some individuals of *Thalassoma hardwicke* held in an aquarium use an anvil to smash a large pellet of food into more manageable pieces^15^. Interestingly, these tool use-like behaviors are observed uniquely in the group of wrasses (the family of *Labridae*) in teleosts. Thus, the brain anatomy of wrasses and their close relatives is of great interest to understand the evolution of tool-using behavior and its related higher-order cognitive functions.

Our developmental studies have shown that the telencephalon, hypothalamus, and sensory nuclei of teleosts^16–20^ differ greatly from amniotes. Indeed, teleost brain organization appears to be much less conserved than previously thought. Notably, teleosts possess a remarkable ventral structure called the inferior lobe, which is absent in tetrapod brains and whose functions remain largely unknown^21^. At a gross morphological level, the ventral parts of the teleost brain appear much more developed in comparison to amniote brains.

These observations raise the question of how encephalization occurred in the teleost lineage, and how the brains of teleosts with remarkable cognitive abilities, such as wrasses, may differ from other species. Is the teleost pallium the brain center that is responsible for higher-order cognitive functions, similar to the amniote brain?

In order to uncover which brain structures are expanded in teleost species demonstrating complex behavioral repertoires, we examined the cellular composition of their major brain regions and compared them with other teleost species located at various phylogenetic positions. We also compared the connectivity of the pallium in encephalized and poorly encephalized species. Our quantitative and qualitative study revealed that, unlike in amniotes, the ventral part of the brain including the inferior lobe is significantly developed in the brains of tool-using species, in which the inferior lobe is heavily connected to the pallium. This illustrates how encephalization in teleosts and amniotes followed different evolutionary paths.

## Results

### The tool-using wrasse *Choerodon anchorago* has more cells in its brain than a hamster twice its body mass

**Fig. 1:**
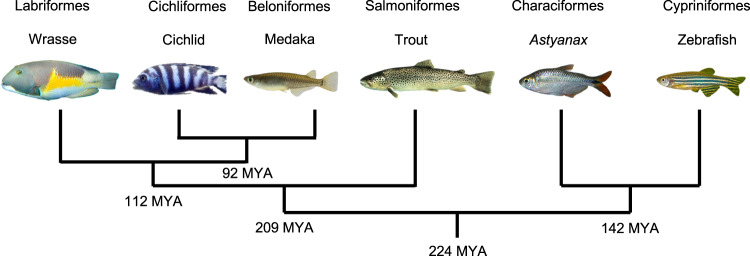
Phylogenetic tree of the teleost species sampled in this study. In this study, we refer to the medaka, trout, *Astyanax*, and zebrafish as the “outgroup”. Numbers at the root of each tree branches represent the estimated time of divergence (MYA: million years ago). The last common ancestor of these species can be traced back to 224 million years ago (http://www.timetree.org^84^).

The body mass and brain mass were measured for 11 species of teleost: a group of three wrasse species (*Choerodon anchorago*, *Labroides dimidiatus, Thalassoma hardwicke*), a group of four cichlid species (*Maylandia zebra*, *Neolamprologus brichardi*, *Ophthalmotilapia boops*, *Amatitlania nigrofasciata*), and a group of four other species (the medaka (*Oryzias latipes*), zebrafish (*Danio rerio*), *Astyanax* surface fish (*Astyanax mexicanus*), and trout (*Salmo trutta*), hereafter referred to as the “outgroup”) (Fig. 1, see “Methods” section). Total number of cells in the brain was determined using the isotropic fractionator (see “Methods” section).

Remarkably complex behaviors have been reported in the group of wrasses: tool use in the case of *C. anchorago*^13^ and *T. hardwicke*^15^, and complex social cognition (e.g., altruism and punishment) in the case of *L. dimidiatus*^22–25^. In cichlids, although no instances of tool use have been observed, many species demonstrate complex social interaction with parental care^26–28^, and there are several studies reporting cognitive abilities such as individual recognition, quantity discrimination, and transitive inference^29–33^.

Among the wrasses studied, body mass ranged from 1.55 to 91.52 g, brain mass from 34.62 to 338.8 mg, and total number of cells in the brain from 45.7 to 185.08 million (Table 1). Wrasses were wild caught and were generally young adults (estimated by morphology); however, one large adult of *C. anchorago*, weighing around ten times as much as the other individuals, was also sampled. Excluding this large specimen from the analyses did not affect the statistical significance of the observed results, so we included it in the main analyses presented here (Supplementary Figs. 1–3 show the data excluding the large specimen; see also Table 1, Supplementary Table 1, and Supplementary File 1). In cichlids, body mass ranged from 5.15 to 20.28 g, brain mass from 42.43 to 96.94 mg and total number of cells in the brain from 37.54 to 61.78 million (Table 1). In the “outgroup”, body mass ranged from 0.492 to 177.15 g, brain mass from 8.38 to 354.73 mg, and total number of cells in the brain from 6.66 to 100.84 million (Table 1). Overall, the specimens used in this study were rather small, and future studies using larger indidivuals would be useful to confirm our results.

**Table 1:** Cellular composition of the brains of 11 teleost species.

Species	*n*	Body mass (g)	Brain mass (mg)	Total cells (x10^6^)
Wrasses				
* Labroides dimidiatus*	3	1.55 ± 0.35	34.62 ± 5.62	45.7 ± 6.67
* Thalassoma hardwicke*	3	12.07 ± 4.14	132.82 ± 23.49	116.78 ± 25.62
* Choerodon anchorago*	4	91.52 ± 137.39	338.80 ± 206.95	185.08 ± 78.73
Choerodon anchorago*	3	22.85 ± 4.54	235.44 ± 11.67	146.11 ± 13.59
Cichlids				
* Neolamprologus brichardi*	5	5.15 ± 1.53	42.43 ± 4.26	37.54 ± 7.08
* Amatitlania nigrofasciata*	5	20.28 ± 7.85	75.05 ± 12.4	56.62 ± 6.37
* Opthalmotilapia boops*	3	7.04 ± 2.39	80.32 ± 7.27	56.37 ± 6.35
* Maylandia zebra*	3	14.59 ± 2.58	96.94 ± 6.62	61.78 ± 3.72
Others				
* Oryzias latipes*	5	0.492 ± 0.07	8.38 ± 1.12	6.66 ± 0.54
* Danio rerio*	5	0.73 ± 0.21	9.74 ± 0.2	8.92 ± 0.69
* Astyanax mexicanus*	5	3.7 ± 1.17	43.55 ± 4.35	26.09 ± 2.74
* Salmo trutta*	4	177.15 ± 45.05	354.73 ± 33.16	100.84 ± 8.58

Choerodon anchorago* data when a large individual of *Choerodon anchorago* is excluded.

All values are mean ± SD.

**Fig. 2:**
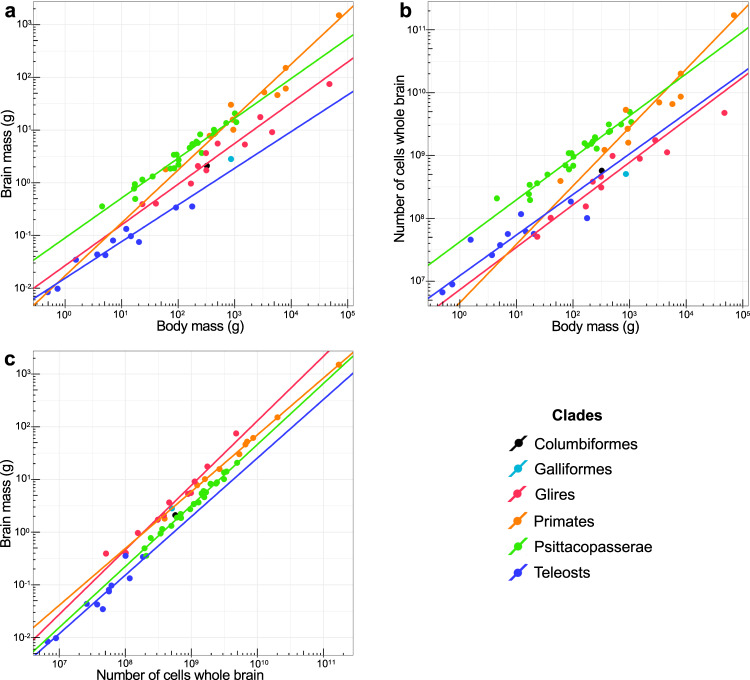
Teleosts have small, cell-dense brains that contain more cells than the brains of rodents of similar body mass. The fitted reduced major axis (RMA) regression lines are displayed only for correlations that are significant. Each point represents the mean value of a species. X and y axes are in log_10_ scales. All regression lines are significantly different, except for the regression lines of Glires and Primates in (**c**). **a** Brain mass plotted as a function of body mass. Teleosts have smaller brains than birds and mammals of similar body mass. **b** Total number of cells in the brain plotted as a function of body mass. Teleost brains contain less cells than bird and primate brains, but more cells than the brains of rodents of similar body mass. **c** Brain mass plotted as a function of total number of cells in the brain. Cellular density inside the teleost brain is higher than in birds and mammals. Columbiformes include pigeons, Galliformes include chickens, Glires include rodents, Psittacopasserae include Passeriformes (songbirds) and Psittaciformes (parrots). See also Table 1. For statistics, see «Methods» section.

Compared to previously published data, teleosts have smaller brains than birds, primates or rodents of similar body mass (Fig. 2a). By contrast, teleost brains contain more cells than the brains of rodents of similar body mass, albeit not as many as birds and primates, (Fig. 2b). For instance, the brain of the tool-using wrasse *C. anchorago* contains on average more cells than the brain of the nearly two times heavier hamster (*Cricetus cricetus*).

Cellular density inside the teleost brain is higher than in birds and mammals, with teleosts having as many cells as rodent brains more than four times larger (Fig. 2c). For example, the large *C. anchorago* individual sampled had 301.9 million cells in its brain, nearly as many cells as a rat (*Rattus norvegicus*), even though its brain is 2.6 times lighter.

### Encephalization and relative mass or number of cells in the telencephalon of teleosts are not correlated

**Fig. 3:**
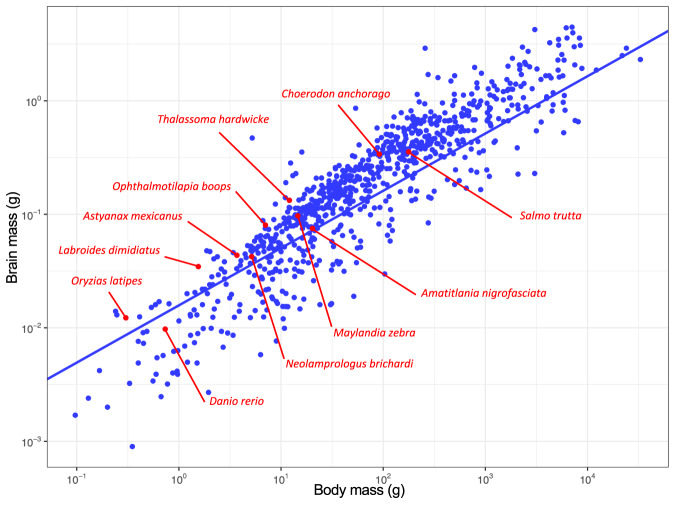
Encephalization in 11 species of teleosts (red) compared to a large dataset of actinopterygians (blue). Brain mass is plotted as a function of body mass, and the phylogenetically corrected (phylogenetically generalized least squares regression test, PGLS) allometric line is shown. Each point represents the mean value of a species. X and y axes are in log_10_ scales. The phylogenetic regression slope for actinopterygians is of 0.50 ± 0.01. Adjusted R^2^: 0.8382, *t* = 65.978, *p* < 0.0001. See also Supplementary Table 1 and «Methods» section.

Residuals obtained by fitting a log_10_-log_10_ regression of brain mass against body mass data from this dataset with previously published data on actinopterygians (Fig. 3) using a phylogenetic generalized least square (PGLS) model ranged from −0.142 (*D. rerio*) to 0.38 (the wrasse *T. hardwicke*), with only one other species (*C. anchorago*) with a residual >0.30 (Fig. 3, Supplementary Table 1). Excluding the large *C. anchorago* from our analysis gave a residual of 0.488 for *C. anchorago*, placing it above *T. hardwicke* (Supplementary Table 1, see Supplementary File 1). Overall, these two tool-using species were the most encephalized of our dataset.

**Fig. 4:**
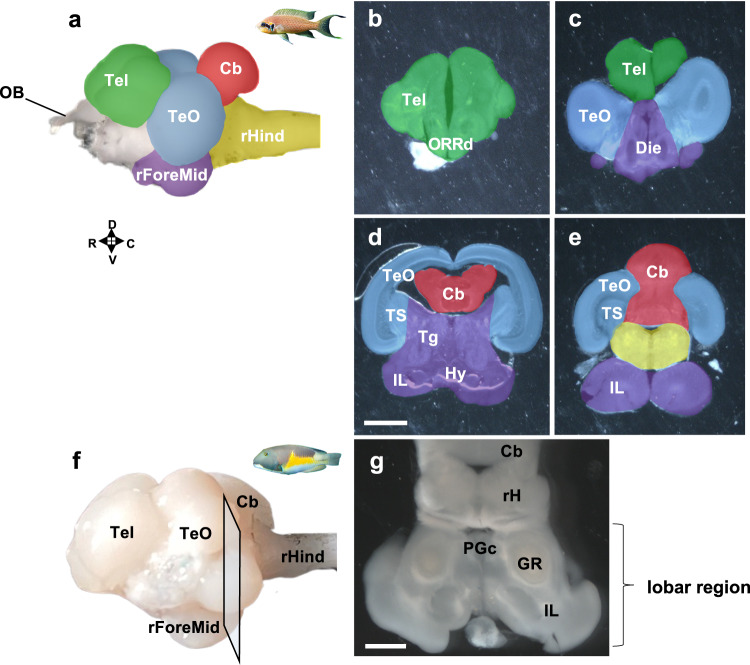
Illustration of brain structures of teleosts. **a**–**e** Dissection of the five major structures for the isotropic fractionator visualized on the brain of the cichlid *Neolamprologus brichardi*. **a** Lateral external view of the brain of the cichlid *Neolamprologus brichardi*. The different brain regions are color-coded. The uncolored regions are the olfactory bulbs and cranial nerves. **b**–**e** 300 µm frontal sections of the brain of *Neolamprologus brichardi* from rostral to caudal, showing the boundaries of the five major brain regions. The regions are highlighted following the color code in (**a**). **f**, **g** Illustration of the lobar region of the brain of the wrasse *Choerodon anchorago*. **f** Lateral external view of the brain of the wrasse *Choerodon anchorago* indicating the level of the frontal section shown in (**g**). We collectively refer to the area containing the PGc, GR, and inferior lobe as the lobar region, which is a teleost-specific structure absent in the tetrapod brain. Brain regions: Cb cerebellum, Die diencephalon, GR corpus glomerulosum pars rotunda, Hy hypothalamus, IL inferior lobe, ORRd dorsal optic recess region, PGc preglomerular nucleus pars commisuralis, rForeMid: rest of the forebrain/midbrain, rHind rest of the hindrain, Tel telencephalon, Tg tegmentum, TeO optic tectum, TS torus semicircularis. Scale bars: 1 mm. R: rostral; C: caudal; D: dorsal; V: ventral. See also «Methods» section.

In order to compare the degree of encephalization with the relative mass and cellular composition of major brain regions, the brains of ten species were dissected into five parts (Fig. 4a): the telencephalon (Tel), the optic tectum (TeO), the rest of the Forebrain/Midbrain (rForeMid; which includes the inferior lobe), the cerebellum (Cb) and the rest of the Hindbrain (rHind) following the rostro-caudal and dorso-ventral axis (Fig. 4b–e, See “Methods” section). These structures were weighed and the number of cells contained in each structure was determined using the isotropic fractionator^34^. No statistically significant correlations were found between encephalization and the relative mass and relative number of cells of the Tel, TeO, rForeMid, Cb (Supplementary Fig. 4a–d). The only structure that showed a statistically significant correlation with encephalization was the rHind (Supplementary Fig. 4e), with a negative correlation for both the relative mass and relative number of cells (See Methods). This indicates that more encephalized species of teleosts have a relatively smaller rHind containing a relatively smaller number of cells.

These results suggest that teleost brains have evolved very differently from amniote brains. Unlike in mammals and birds, encephalized species of teleosts don’t have an extremely large Tel.

### Wrasses have a relatively larger Tel and rForeMid, but not a larger relative number of cells compared to other teleosts

**Fig. 5:**
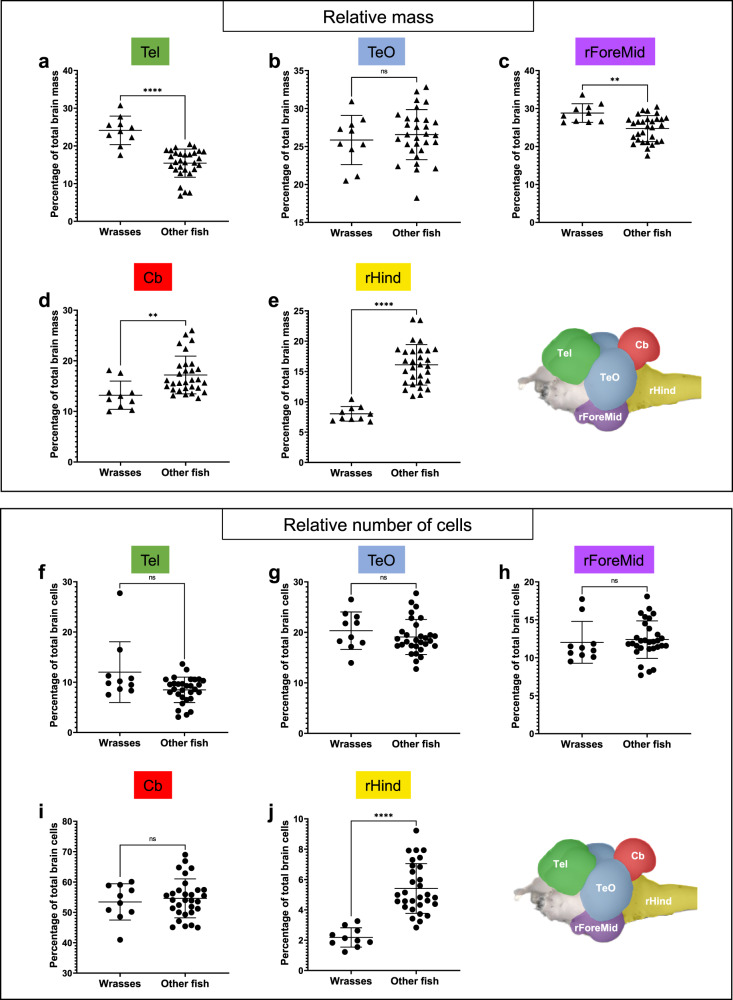
Relatively larger Tel and rForeMid without a corresponding increase in the relative number of cells in wrasses compared to other teleosts. Three species of wrasses (« Wrasses »: *Choerodon anchorago*, *Labroides dimidiatus*, *Thalassoma hardwicke*) were compared with seven other teleost species from various orders (« Other fish »: *Astyanax* mexicanus, *Amatitlania nigrofasciata, Danio rerio, Maylandia zebra, Neolamprologus brichardi, Ophtalmotilapia boops, Salmo* trutta). Top panel (**a**–**e**): Comparison of the relative mass of the Tel (**a**), TeO (**b**), rForeMid (**c**), Cb (**d**), and rHind (**e**). Wrasses have a relatively larger Tel and rForeMid compared to other teleosts. Bottom panel (**f**–**j**): Comparison of the relative number of cells in the Tel (**f**), TeO (**g**), rForeMid (**h**), Cb (**i**), and rHind (**j**). Despite having a relatively larger Tel and rForeMid, wrasses don’t have a larger proportion of cells in those structures compared to other teleosts. Statistical analysis: Mann-Whitney’s test. Each point represents individual values. Error bars: mean ± SD. ns: non significant, **p < 0.01, ****p < 0.0001. Brain regions: Cb: cerebellum, rForeMid: rest of the forebrain/midbrain; rHind: rest of the hindrain; Tel: telencephalon; TeO: optic tectum. For statistics, see «Methods» section.

Comparing species in a one-to-one manner didn’t reveal any consistent differences in either the relative mass or relative number of cells across structures (Supplementary Fig. 5, See Methods). However, a trend towards larger Tel and rForeMid was observed when examining wrasses as a whole (Supplementary Fig. 5). Wrasses have large brains and display the most flexible behavioral repertoires, including tool use. We thus aimed to investigate any differences in their brain morphology compared to the other teleosts. To this end, the relative mass and number of cells in the five major regions of the brains of all wrasse species were compared with those of the other species of teleosts sampled in this study (Fig. 5).

The relative mass of the Tel and rForeMid was significantly higher in wrasses compared to other teleosts, with the Tel accounting for 24.11% ± 3.78% of total brain mass in wrasses compared to 15.43% ± 3.74% in other species (Fig. 5a). While the Tel in wrasses is larger than in other teleosts, it remains modest when compared to amniotes. The rForeMid accounted for 28.82% ± 2.46% of total brain mass in wrasses compared to 24.72% ± 3.43% in other species (Fig. 5c). The relative mass of the Cb and rHind were significantly lower in wrasses compared to other teleosts (Fig. 5d, e). No significant difference was found in the relative mass of the TeO between the two groups (Fig. 5b).

Despite the larger size of the Tel and rForeMid in wrasses, isotropic fractionator data revealed that there is no significant difference in the relative number of cells in these two structures compared to other species. The Tel accounted for 12.02% ± 6.04% of total brain cells in wrasses compared to 8.49% ± 2.54% in other species (Fig. 5f), while the rForeMid accounted for 12.04% ± 2.76% of total brain cells in wrasses and 12.39% ± 2.47% in other species (Fig. 5h). No significant difference in relative number of cells was found in either the Cb (Fig. 5i) or TeO (Fig. 5g), whereas the rHind (Fig. 5j) accounted for a significantly smaller relative number of cells in wrasses compared to other species (2.18% ± 0.64% and 5.41% ± 1.64%, respectively).

Similar results were obtained when comparing cichlids to the “outgroup” (Supplementary Fig. 6, See «Methods» section). Cichlids had a slightly larger relative number of cells in the rForeMid compared to the “outgroup”, but there was no significant difference with wrasses.

Overall, these results show that wrasses have a relatively larger Tel and rForeMid compared to other teleosts. However, these two structures do not contain a larger proportion of cells than in other teleosts.

### Pallium and inferior lobe display increased connectivity in wrasses compared to other teleosts

**Fig. 6:**
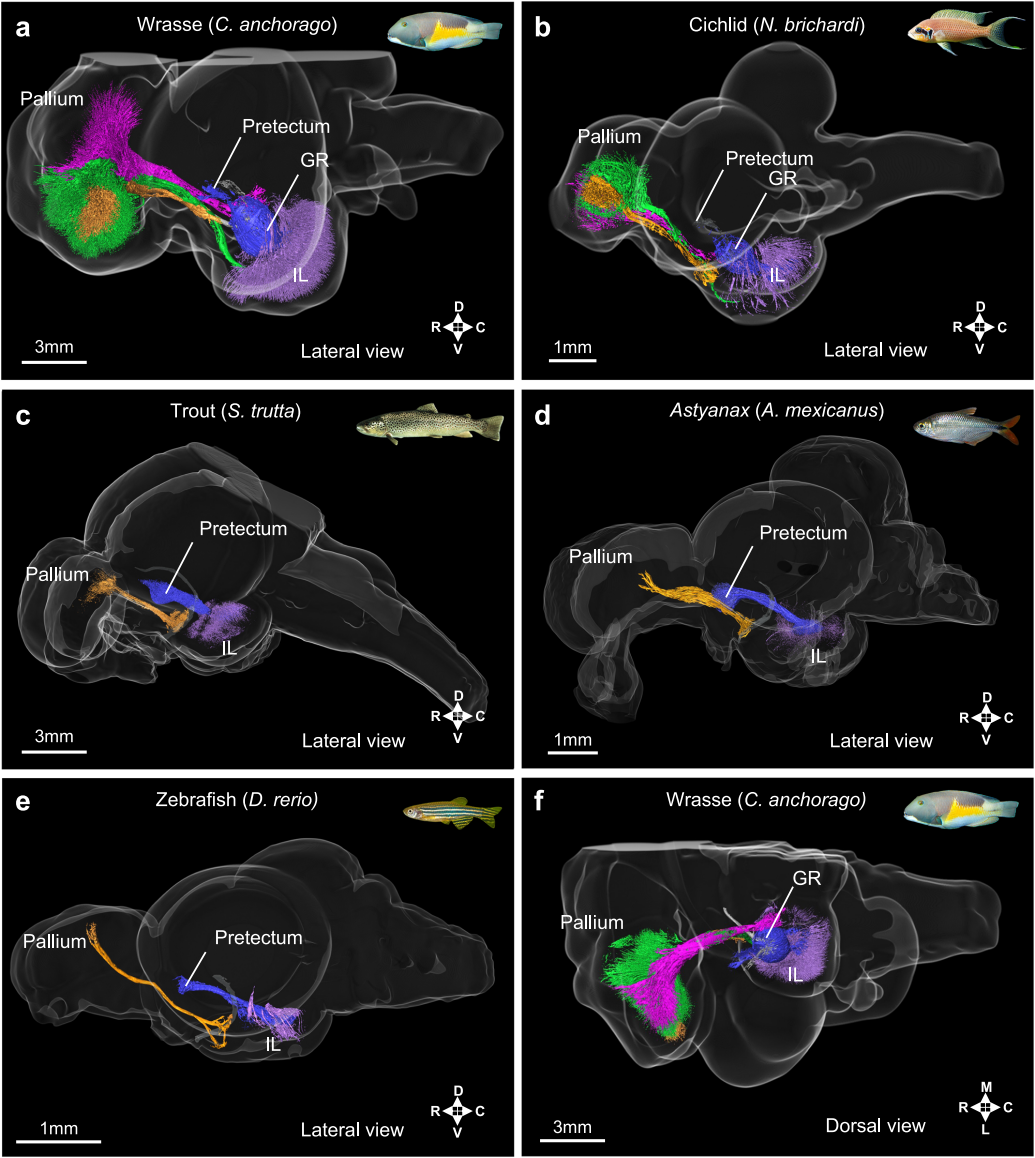
The pallio-lobar tracts are massively enlarged in the wrasse and cichlid, while they are absent in the trout, the *Astyanax* surface fish and the zebrafish. 3D selective visualization of inferior lobe fiber tracts comparing the wrasse (*C. anchorago*; **a**), the cichlid (*N. brichardi*; **b**), the trout (*S. trutta*; **c**), the *Astyanax* surface fish (*A. mexicanus*; **d**), and the zebrafish (*D*. rerio; **e**). Lateral views are shown in (**a**–**e**), while a dorsal view of one side of the wrasse brain is shown in (**f**). Homologous tracts are shown in the same color across species. Besides wrasses and cichlids, no fibers connecting the pallium to the inferior lobe were detected in the other species of teleosts examined, irrespective of brain size. The main connections of the inferior lobe in these species are with the pretectum (blue), whereas they are with the pallium in wrasses and cichlids (ventral tract in green, dorsal tract in magenta). Local inferior lobe networks are shown in purple, and preglomerular complex projections to the pallium are shown in orange. Brain regions: GR:corpus glomerulosum pars rotunda, IL inferior lobe. R:rostral, C:caudal; D: dorsal; V: ventral; L: lateral ; M: medial.

We hypothesized that the increase in mass observed in the Tel and rForeMid of wrasses is due to an increase in the neuropil of these structures. To verify this hypothesis, we performed selective visualization of the fibers in the Tel and rForeMid. Whole brains of the wrasse *C. anchorago*, the cichlid *N. brichardi*, the trout *S. trutta*, the *Astyanax* surface fish *A. mexicanus*, and the zebrafish *D. rerio* were cleared, stained with DiI, and imaged on a light-sheet microscope (Fig. 6, Supplementary Movies 1–5, See «Methods» section).

3D reconstruction of the DiI-stained fibers in the Tel and rForeMid of wrasses and cichlids revealed the presence of enriched fiber labeling in the Tel and rForeMid. Most of the inferior lobe, the ventral-most part of the rForeMid, exhibited high fiber density in wrasses (Fig. 6a, Supplementary Movie 1; in purple), while fiber labeling was sparse in the other species examined (trout, Fig. 6c, Supplementary Movie 2; *Astyanax* surface fish, Fig. 6d, Supplementary Movie 3; zebrafish, Fig. 6e, Supplementary Movie 4; in purple).

The telencephalic fibers in the wrasse almost completely occupy the entire pallium. These fibers converge onto the lateral forebrain bundle as they exit the telencephalon and then split again into two major tracts (Fig. 6a, Supplementary Movie 1; in green and magenta). These connect the pallium and the structures in and around the inferior lobe (in an area which we refer to as the lobar region, Fig. 4f,g), and we thus refer to these two tracts as “pallio-lobar tracts”. The ventrally located tract (Fig. 6a, Supplementary Movie 1; in green) directly connects the pallium and the ventral inferior lobe ipsilaterally. The dorsally located tract (Fig. 6a, Supplementary Movie 1; in magenta) courses near the midline ipsilaterally and connects the pallium with a structure called the nucleus preglomerulosus pars commissuralis (PGc) in the lobar region, as well as sending minute fibers to the inferior lobe that run through the periphery of an oval-shaped structure called the corpus glomerulosum pars rotunda (GR). The same tracts are also present in the cichlid brain, albeit more modestly, with a much smaller fiber arborization in both the pallium and inferior lobe (Fig. 6b, Supplementary Movie 5; in green and magenta). Strikingly, in trout, zebrafish, and *Astyanax* surface fish, these tracts were not detectable, and only minimal arborization was found in the pallium and the inferior lobe (Fig. 6c–e, Supplementary Movies 2–4). Both PGc and GR are absent in those species, indicative of the poor development of their lobar region.

Tract tracing studies using the lipophilic dye NeuroVue, biocytin, and biotinylated dextran amine (BDA molecular weight 3000) confirmed the presence of connectivity between the pallium and the lobar region in the wrasse and cichlid brains (Supplementary Fig. 7, See «Methods» section). Biocytin injections in the telencephalon (Supplementary Fig. 7a, white asterisk) allowed us to identify the direction of the projections. Abundant labeled fibers were found in the inferior lobe (Supplementary Fig. 7b), while very few cell bodies were labeled (Supplementary Fig. 7c, white arrows). This suggests that the majority of projections are descending fibers from the pallium to the inferior lobe, with only few ascending fibers from the inferior lobe to the pallium. These fibers reached the inferior lobe through the ventral branch of the lateral forebrain bundle mentioned above.

While pallio-lobar tracts were not detectable in zebrafish with 3D reconstruction of DiI labeled fibers, biocytin injections into the dorsal telencephalon resulted in labeled fibers in the lateral forebrain bundle and terminal labeling in the inferior lobe of zebrafish. This indicates that pallial connectivity with the inferior lobe is present in this species, albeit to a lesser extent than in wrasses and cichlids.

There are two additional fiber tracts observable in all species examined. One contains projections from the sensory preglomerular complex to the pallium (Fig. 6, Supplementary Movies 1–5; in orange)^16,35^, coursing rostrally and joining the lateral forebrain bundle. The most distinct branch of this tract terminates in the lateral zone of the dorsal telencephalic area (Dl) carrying visual information^16^. In the pallium of wrasses and cichlids, these visual terminals (Fig. 6a, b; orange) are embedded in the arborization of the ventral pallio-lobar tract (Fig. 6a, b; green).

The other tract present in all species is the one connecting the inferior lobe with the pretectum (Fig. 6, Supplementary Movies 1–5; in blue). In the trout, zebrafish, and *Astyanax* surface fish, this is the major tract connecting the inferior lobe with the rostral aspect of the brain. In the wrasse and cichlid brain, the pretectal pathway to the inferior lobe is mediated by the GR (Fig. 6a, Supplementary Movie 1; in blue). This only represents a small proportion of inferior lobe connectivity in those species, as the inferior lobe is also heavily connected with the pallium.

The presence of very developed pallio-lobar tracts is unrelated to absolute or relative brain size, as these tracts were not detectable in the large brained trout. Thus, the large quantity of fibers connecting the inferior lobe and the pallium in wrasses and cichlids appears to be remarkable feature of their brain organization compared to other species.

Overall, the presence of the pallio-lobar tracts and their extreme enlargement in wrasses may thus explain the expansion of their Tel and rForeMid without a corresponding increase in the relative number of cells in those structures. This increase in the relative quantity of fibers in tool-using teleost species also parallels what has been observed in the mammalian telencephalon, where primates have a larger proportion of white matter compared to rodents^36,37^.

## Discussion

Mammals and birds have taken two different trajectories of encephalization that have converged onto a process of “telencephalization”, whereby the telencephalon (and in particular the pallium) becomes massively enlarged in highly encephalized species. Our study shows that this is not the case in teleosts.

Sampling of a phylogenetically broad range of teleost species revealed that encephalization in teleosts does not lead to an enlargement of most of the examined brain regions, both in terms of mass and relative number of cells. That is, there was no single particularly prominent structure in highly encephalized teleosts compared to less encephalized ones. Even in the tool-using species (*C. anchorago*), the telencephalon is of a modest size, representing only 27.8% of total brain mass. This is in stark contrast to amniotes, where the telencephalon makes up 80% of total brain mass in tool-using species of primates, parrots and corvids^8,38^.

Compared to amniotes, the rForeMid of teleosts is remarkably large. In previous amniote studies^8,38^, the brain structures corresponding to rForeMid, TeO and rHind were pooled together and called the “rest of brain” on account of their small relative size compared to the telencephalon and cerebellum. While this “rest of brain” represents merely 10–25% of total brain mass in primates, parrots and corvids^8,38^, it does represent 61.1% in the tool-using wrasse *C. anchorago*. The modest telencephalon and the large “rest of brain” of teleosts, even in relatively highly encephalized tool-using species, indicates that unlike in amniotes, encephalization in teleosts is not a process of telencephalization.

In contrast to these differences, mammalian and teleost brains have in common an increase in white matter volume in tool-using species. In mammals, primates have a significantly higher white matter volume to gray matter volume ratio in the telencephalon compared to rodents^36,37^. However, when compared to the number of neurons, rodents actually have more white matter per neuron than primates^37^. This is in part due to the axonal caliber increasing in rodents as neurons are added to the cortex, while axonal caliber remains constant in primates^39,40^. On top of this, as neurons are added to the cortex, cortical connectivity (the fraction of gray matter neurons connected through the white matter) remains constant in rodents, while it decreases in primates. Such a decrease in connectivity in primates as the network grows is indicative of a small-world network, while the rodent cortex appears to be organized as a uniform network^37,41^.

We found wrasses to have much larger amounts of fibers in both their telencephalon and lobar region compared to other species, due to their very large pallio-lobar tracts. The functional significance of this feature of brain organization in teleosts is unclear. As we could only assess fiber tract volume qualitatively rather than quantitatively, we cannot evaluate white matter per neuron values in teleosts. It would be interesting to know whether the increased volume of white matter in wrasses compared to trouts is due to the axonal caliber of neurons increasing (as in rodents) or rather to the increase in the absolute number of neurons, with axonal caliber remaining similar (as in primates). Similarly, connectomics data in tool-using teleosts would help understand the nature of their network architecture: do wrasses possess a uniform network brain like rodents, or a small-world network brain like primates ?

One limitation of our results describing the cellular composition of the teleost brain is that we were only able to obtain total cell numbers for each structure, and we thus could not distinguish between neuronal and non-neuronal cell numbers like in the rest of the isotropic fractionator literature^8,38^. One interesting finding from these studies is that non-neuronal cell density varies at most by one order of magnitude in amniotes, while neuronal density varies by three orders of magnitude^42^. If this non-neuronal density also holds for teleosts, it would indicate that teleosts have neuronal densities in their pallium higher or similar to those of certain songbirds and parrots^8,38,43^. Future studies in teleosts should help verify this hypothesis and shed more light on the functional consequences of high neuronal densities.

The rForeMid corresponds to the ventral part of the forebrain and midbrain, while the Tel and TeO represent the dorsal parts of the forebrain and midbrain respectively. As the rForeMid is large in teleosts, accounting for a quarter to a third of total brain mass, it appears that teleost brains are a lot more “ventralized” compared to amniote brains.

The lobar region (which includes the inferior lobe, GR, and PGc) in particular appeared to account for most of the rForeMid volume. The inferior lobe was long considered to be of hypothalamic origin and used to be named the “inferior lobe of the hypothalamus” as a result. A recent study^21^ has demonstrated that the developmental origin of the inferior lobe is in fact mainly mesencephalic, while the cell populations surrounding the lateral recess of the hypothalamic ventricle, which represent a small part of the inferior lobe, are of hypothalamic origin. Bloch et al. ^21^ has suggested that in the species with a large inferior lobe, it is mainly this mesencephalic part that becomes enlarged, and not the hypothalamic part.

The inferior lobe is especially enlarged in wrasses and cichlids. The interpretation on the evolution of this structure largely depends on the phylogenetic relationship of these groups. Wrasses and cichlids have been considered as closest relatives, forming the group of “labroids”^44^. However, the divergence time obtained by TIMETREE5 (www.timetree.org), as well as other publications, show that they are relatively close, but not the closest, and that cichlids are in fact closer to medaka^45,46^ (Fig. 1). As the medaka does not appear to have a particularly developed inferior lobe, this raises the possibility that the enlargement of the inferior lobe may have occurred independently in different groups of teleosts.

GR forms the root of the lobar region in wrasses and cichlids. It is considered to have important sensory (especially visual) functions, and to project almost exclusively to the inferior lobe^47–49^. Not only does GR have no homolog in tetrapod brains, it has only evolved in some groups of teleosts^50^. This also appears to be the case for PGc, and we thus consider GR and PGc as specialized nuclei present only in groups of teleosts which possess large connectivity between the pallium and lobar region.

Another structure of the rForeMid that is also involved in sensory processing is the preglomerular complex, which is considered to play a role equivalent to the amniote thalamic nucleus. As our previous study has shown, it is mostly made up of cells of a mesencephalic origin^16^. In that sense, it appears that teleost brains are largely more mesencephalized than amniote brains. Altogether, sensory systems in teleosts and tetrapods are not as conserved as previously thought but have evolved independently in each lineage.

Teleosts thus display marked differences in the organization of their brains compared to amniotes, with mesencephalic structures accounting for a much larger proportion of total brain mass and playing a prominent role in sensory processing.

Our current study revealed that in the wrasse and cichlid brains, the inferior lobe is highly connected with the pallium. This seems to be especially apparent in tool-using species. As some previous studies already suggested^21,51^, this challenges the previous notion that the inferior lobe is merely a food motivation center^52–55^. The inferior lobe receives gustatory information^48,56–58^, and due to its position directly next to the hypothalamus, it was thought to be homologous to the lateral hypothalamus of mammals^55^. Direct electrical stimulation of the inferior lobe resulted in behaviors such as biting on a mirror or snapping at objects in freely moving fish^52,53^, and inferior lobe activation was found during detection of moving objects in larval zebrafish^54^. With the assumption that the inferior lobe was homologous to the mammalian hypothalamus, these functional data have been interpreted as the inferior lobe playing a role in feeding behaviors. However, since this previous view of inferior lobe homology has been shown to be erroneous^21^, a reinterpretation of this data is necessary.

**Fig. 7:**
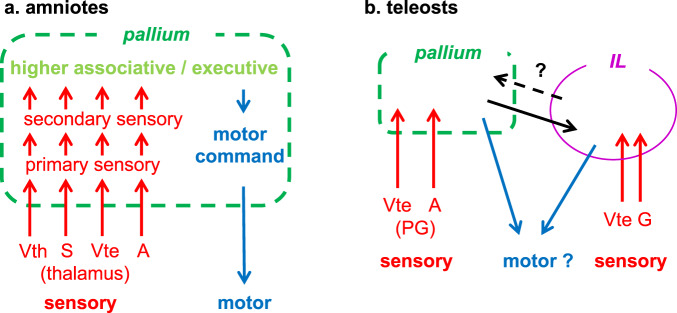
Comparison of functional connectivity in relation to sensory inputs and motor outputs in amniotes and teleosts. **a** Simplified diagram showing input/output connectivity of the pallium commonly found in mammals and birds (analogous, not necessarily homologous). Sensory inputs are shown in red, while motor outputs are shown in blue. The primary sensory areas in the pallium receive modal-specific sensory inputs from subtelencephalic sensory nuclei, mainly through the thalamus in the case of tetrapods. Note that there are two major visual pathways terminating in the pallium both in mammals and birds. The diagram is modified from Yamamoto and Bloch (2017)^17^. **b** Simplified diagram showing input/output connectivity of the pallium and inferior lobe (IL) in teleosts. The sensory afferents to the pallium in teleosts are mainly mediated via the preglomerular complex (PG) instead of the thalamus. In addition to the pallium, the inferior lobe receives sensory inputs of different modalities, here showing only visual and gustatory, which are the dominant ones. The pallium and the inferior lobe are highly connected in some teleost groups such as wrasses and cichlids. Sensory modalites: A: auditory, G: gustatory, S: somatosensory, Vte: visual (tectofugal), Vth: visual (thalamofugal).

In addition to gustatory inputs, the inferior lobe receives visual inputs from the TeO via the pretectum^47–49,54^. In the species where GR is present, it has been suggested that the inferior lobe also receives auditory^47^ and somatosensory^49^ information. As a result, the inferior lobe has also been proposed to be a multi-sensory integration center^48,49,57,59^. In addition, since its main output is to the lateral valvular nucleus, which projects to the cerebellum^48,60^, its functions may be motor-related. This sensory input and motor output connectivity pattern in the inferior lobe is rather similar to what has been found in the amniote pallium. As the teleost pallium itself receives sensory information of different modalities (e.g., auditory and visual inputs via the preglomerular complex), the inferior lobe seems to serve as another sensory integration center in the teleost brain (Fig. 7).

The presence of multimodal inputs to the inferior lobe is likely to be a common feature in teleosts, but the particularity of the wrasse and cichlid brains is the inferior lobe’s intense connectivity with the pallium. The major connectivity of the inferior lobe of other fish like trout, *Astyanax* surface fish, and zebrafish is with the pretectum, which is involved in stereotyped movements such as the optokinetic response^61,62^ or the prey detection J-turn in larval zebrafish^63^. Those types of movements are sufficient for simple foraging behaviors without flexibility. It is then possible that the elaborated connectivity with the pallium present in wrasses and cichlids may have allowed for the emergence of their complex behavioral repertoire. The large gustatory inputs of the inferior lobe may for instance be involved in different functions than simply eating in these species.

As fish do not have hands, they use their mouth to manipulate objects, and could likely have fine discriminative touch and motor control via the lips and oral cavity, functions which could involve the inferior lobe. Apart from tool use in wrasses, cichlids display object play and elaborate nest building behaviors^64–66^, which also require this kind of precise motor control. Thus, one possibility is that the inferior lobe plays a role in motor cortex-like functions. Hodological data showing that the inferior lobe receives descending projections from the pallium (Supplementary Fig. 7) and projects to the lateral valvular nucleus projecting to the cerebellum^49,60^ rather support the idea that the inferior lobe is involved in a motor aspect.

Another possibility is that the inferior lobe may also be a part of the higher-order areas, like the executive area (prefrontal cortex-like area). The presence of a higher-order association center in the teleost pallium has hardly been investigated so far. In mammals and birds, the sensory association areas are located in the periphery of the primary sensory areas, and project to the executive area^67–72^. If the teleost association areas are organized in the same manner, the area where the arborization of the ventral pallio-lobar tract is located (Fig. 6a, b; green) would be a good candidate for the visual association area. The dorsal telencephalic area (Dl), the putative teleost primary visual area (Fig. 6a, b; orange pallial arborizations), projects to the surrounding pallial areas including the central part of the pallium^35,73^, which in turn project to the IL. This projection pattern is similar to the “primary sensory → sensory association → executive“ pattern observed in amniotes.

In any case, the ability to use tools requires both fine motor skills and executive functions (e.g., long working memory). These functions should reside in the pallium and/or inferior lobe of wrasses, unless this tool use by wrasses is a stereotyped behavior and not context dependent flexibility, in which case a large executive area would not be required. Additional connectivity and functional studies are required to verify how higher-order areas are organized in the teleost brain.

In conclusion, our findings revealed that the encephalization process in teleosts is different from what has previously been described in amniotes. While the pallium also appears to be important for higher-order cognitive functions in teleosts, the large pallio-lobar tracts in the tool-using fishes demonstrate the functional importance of the inferior lobe in relation to the pallium, which may be critical for such complex behaviors. Since the inferior lobe has no homolog in amniotes, at least three different brain organizations enabling higher-order cognitive functions may have evolved independently in mammals, birds and teleosts.

## Methods

### Study animals and brain sampling

11 species of teleost were examined: a group of 3 wrasse species (*Choerodon anchorago*, *Labroides dimidiatus, Thalassoma hardwicke*), for which complex behaviors (tool use and social cognition) have been reported, 4 cichlid species (*Maylandia zebra*, *Neolamprologus brichardi*, *Ophthalmotilapia boops*, *Amatitlania nigrofasciata*), which are capable to a lesser extent of complex behaviors, and a group of 4 other species (the medaka (*Oryzias latipes*), zebrafish (*Danio rerio*), *Astyanax* surface fish (*Astyanax mexicanus*), and trout (*Salmo trutta*)).

Adult individuals of zebrafish (*Danio rerio*), medaka (*Oryzia latipes*) and *Astyanax mexicanus* were obtained from the animal facility in NeuroPSI (Saclay, France). Adult trouts (*Salmo trutta*) were sourced from the animal facility at INRAE (Jouy-en-Josas, France). *Neolamprologus brichardi*, *Amatitlania nigrofasciata* and *Danio rerio* individuals used for tract-tracing with BDA and biocytin were obtained from local dealers in Japan.

Sexually mature individuals of both sexes of wrasse and cichlid species were sourced from commercial providers (*Choerodon anchorago*, *Labroides dimidiatus*, *Thalassoma hardwicke*: Marine Life (Paris, France); *Maylandia zebra* and *Ophthalmotilapia boops*: Abysses (Boissy-Saint-Léger, France); *Amatitlania nigrofasciata*: Abysses, Aquariofil.com (Nîmes, France), *Neolamprologus brichardi*: Abysses, Aquariofil.com). Wrasses were wild caught and tended to be young adults, but one large adult of *Choerodon anchorago* weighing around ten times as much as the other individuals was also sampled. Statistical analysis showed that the data from this large individual did not impact the statistical significance of our results (see Supplementary File 1).

Zebrafish and medaka specimens were anesthetized and euthanized in ice-cold water, weighed on a precision scale and fixed in ice-cold 4% paraformaldehyde (PFA) in 0.01 M phosphated buffer saline containing 0.1% Tween 20 (PBST). All other fish specimens were euthanized by an overdose of MS222, weighed, and immediately perfused transcardially with 4% PFA in PBS. 24 h post-fixation, brains were dissected, weighed on a precision scale, and kept in 4% PFA in PBS for another 24 h before being transferred in anti-freeze solution (30% glycerol, 30% ethylene glycol, 30% H_2_0, 10% PBS 10X) and kept at −20 °C for later use. Brains used for NeuroVue tract-tracing were kept in 4% PFA at 4 °C until use.

All procedures were conducted in compliance with the official regulatory standards of the French Government and in compliance with the official Japanese regulations for research on animal, and the regulations on Animal Experiments in Nagoya University.

### Isotropic fractionator

The medaka brains (*n* = 5) were left undissected.

The brains of *n* = 5 individuals of each species, except the trout (*n* = 4), *M. zebra* (*n* = 3), *C. anchorago* (*n* = 4), *L. dimidiatus* (n = 3), *T. hardwicke* (*n* = 3) and *O. boops* (*n* = 3) were rinsed in PBS and embedded in 3% agarose containing 1% Tween 20 and sectioned at 300 µm in the frontal plane with a vibratome (Leica VT 1200 S). Under a stereomicroscope (Olympus SZX7), the brain was manually dissected using a microsurgical knife into five regions following the rostro-caudal and dorso-ventral axis (Fig. 4).

The dorsal part of the secondary prosencephalon, which includes the telencephalon and the dorso-rostral part of the optic recess region (ORR)^17,18^, was excised. This region was labeled “telencephalon” (Tel). The second region dissected was the dorsal part of the mesencephalon, which includes the tectum opticum and the torus semicircularis and was labeled “optic tectum” (TeO). The third region included the ventral part of the secondary prosencephalon (i.e., the hypothalamus), the diencephalon and the ventral part of the mesencephalon (i.e., the tegmentum and the inferior lobe) and was labeled “rest of the forebrain/midbrain” (rForeMid). The fourth excised region was the dorsal part of the rhombencephalon (i.e., the cerebellum) (Cb). Finally, all the other hindbrain structures, including the medulla oblongata, were labeled “rest of the hindbrain” (rHind). Sections were dried with a paper towel, weighed on a precision scale and kept in 4% PFA for later use.

The number of cells in the five main regions of the teleost brain was determined using the isotropic fractionator method^34^. This method produces results similar to unbiased stereology^74^. Each structure was manually homogenized in 40 mM sodium citrate with 1% Triton X-100 using a Tenbroeck tissue grinder (Ningbo Ja-Hely Technology Co., Ningbo, China). Once an isotropic suspension of isolated cell nuclei was obtained, the suspension was then centrifuged, and the supernatant was collected. The cell nuclei in both the suspension pellet and the supernatant were stained by adding PBS with 1% diamino-phenyl-indol (DAPI). Additionally, a predetermined volume of PBS was added to the suspension to adjust the nuclei density for counting.

To determine the total number of cells in the tissue, four 10 µL aliquots of the suspension and of the supernatant were counted under an epifluorescence microscope (Axio Imager, Zeiss) with X200 magnification using a Blaubrand Malassez counting chamber (Brand Gmbh, Wertheim, Germany). Mean nuclear density in the suspension and the supernatant was multiplied by their total volume and added up to determine the total number of cells in the brain tissue.

To determine the total number of neurons in each sample, we initially aimed at performing an anti-NeuN immunoreaction in PBS using anti-NeuN antibodies. However, after testing multiple antibodies (anti-NeuN rabbit Antibody, ABN78 & ABN78C3, Merck; anti-NeuN rabbit Antibody, ab177487, Abcam; anti-NeuN mouse Antibody, MAB377, Merck) and increasing antibody concentrations (up to 1:50), we were unable to obtain reliable neuronal nuclear staining. Further tests on brain sections also failed to label teleost neuronal nuclei with NeuN in a consistent manner, suggesting that this tool was not appropriate for teleost tissues. Consequently, we decided to present data on total cell numbers for our brain samples.

### Whole-brain clearing and fiber staining

Lipophilic dye was applied to *n* = 2 whole brains of *D. rerio*, *A. mexicanus*, *N. brichardi*, *C. anchorago* and *S. trutta* for fiber bundles tracing.

Brains stored in anti-freeze solution at −20 °C were washed with PBST for at least 1 day. Samples were bleached for 2 h under intense lighting (>10,000 lux, GVL-SPOT-50-FIXV4-230VAC, GreenVisuaLED) in a fresh depigmentation solution of 5% H_2_O_2_, 0.05% sodium azide in PBS. The bleached samples were thoroughly washed with PBST overnight and were then subjected to a size-dependent delipidation step in CUBIC-L^75^. They were first immersed in a mixture of 50% PBST/50% CUBIC-L overnight under gentle shaking followed by an incubation in CUBIC-L at 37 °C under agitation for 1–2 days for *D. rerio*, 3 days for *A. mexicanus*, 4 days for *N. brichardi* and 6 days for *C. anchorago* and *S. trutta* with solution renewed once. Delipidated specimens were washed with PBST for at least 4 h prior to staining.

Staining was performed in solutions that were originally designed for immunostaining of zebrafish larvae^76^. Samples were immersed in a blocking solution of 10% normal goat serum, 10% DMSO, 5% 1 M PBS-glycine, 0.5% Triton X-100, 0.1% sodium deoxycholate, 0.1% IGEPAL CA-630 and 0.1% saponin in PBST overnight at 37 °C under gentle shaking. Subsequently, specimens were stained with 2 µg/ml of DiI (D282) in a solution of 2% NGS, 20% DMSO, 0.05% sodium azide, 0.2% Triton-X100, 10 µg/mL heparin and 0.1% saponin at 37 °C under rotation for a specimen-dependent duration.

After a last washing step in PBST, refractive index matching was carried out in weakly basic CUBIC-R solution^75^. Brains were soaked in a mixture of 50% PBST/50% CUBIC-R overnight under agitation and then kept in CUBIC-R (refractive index = 1.52) prior to mounting.

### Whole-brain 3D imaging

Refractive index matched samples were embedded in a filtered (pore size 5.0 µm) melted agarose solution containing 2% agarose, 70% CUBIC-R in distilled H_2_O. CUBIC/agarose gels were immersed in CUBIC-R at room temperature for a minimum of 1 day to homogenize refractive indices.

Images were acquired with two commercial light-sheet fluorescence microscopes. Acquisitions were performed with an Ultramicroscope II (Miltenyi Biotec) using a 1.1x NA 0.1 MI PLAN objective and a DC57 WD17 0 dipping cap coupled to a 2x magnification lens, or a LVMI-Fluor 4x/0.3 WD6 objective without additional magnification. A Lightsheet 7 (Zeiss) equipped with 10 × 0.2 foc illumination and 5 × 0.16 foc detection optics was also used. According to their size and the type of microscope images were acquired from dorsal or sagittal view. Cotton seed oil was poured on the surface of the imaging medium as an impermeable layer to avoid evaporation-induced refractive index changes during imaging. 16-bit images were acquired by a pco.edge 5.5 sCMOS camera (2560 × 2160 pixels, pixel size 6.5 µm × 6.5 µm) on the Ultramicroscope II or a pco.edge 4.2 sCMOS camera (1920 × 1920 pixels, pixel size 6.5 µm × 6.5 µm) on the Lightsheet 7, following sample excitation with laser 488 and 561 nm. The z-step size was fixed to 6 µm on the Ultramicroscope II and 5.176 µm on the Lightsheet 7, which represents nearly half of the theoretical lightsheet thickness.

### 3D image reconstruction and manual segmentation

For the inter-species comparison of the anatomy of the tracts connecting the Tel with the rForeMid, these structures were segmented manually using the 3D visualization and reconstruction software Amira 2019 (Thermo Fisher Scientific).

The combination of the overall size of the specimens and the required resolution/voxel size demanded tiled image acquisition. The resulting image stacks were merged using the Grid/Collection stitching plugin^77^ in Fiji.

In preparation for the manual segmentation, the signal-to-noise ratio of the merged data was improved by subtracting the gaussian noise (Fiji, Gaussian Blur 3D, Kernel 10,10,10) from the original data. After manual segmentation of the original data and the denoised data by an unbiased researcher, the defined regions were refined by multiplying the denoised data with the individual binary masks of the segmentations.

The 3D reconstructions in Fig. 6 were produced on *n* = 2 brains for each species by selective visualization of the denoised features under investigation in this study within the framework of the overall anatomy of the corresponding brains.

### Tract-tracing with NeuroVue

In order to confirm the presence of the inferior lobe fiber tracts visualized with DiI staining, tract-tracing experiments were performed using NeuroVue (Polysciences), a lipophilic dye which allows both retrograde and anterograde tracing and can be used on fixed brain tissue^78^. Small triangular pieces of NeuroVue filter paper were inserted into the inferior lobe of *n* = 3 specimens of *A. mexicanus*, *N. brichardi* and *C. anchorago*, and into the pallium of *n* = 3 specimens of *N. brichardi*. Brains were then incubated at 36 °C in 4% PFA in PBS for 4 (*A. mexicanus*) to 12 days (*C. anchorago*).

Following incubation, 80 µm sections were cut with a vibratome (Leica VT1200S), both in frontal and sagittal planes in order to visualize the fiber tracts’ orientation in 3D. Sections were then treated with DAPI before being mounted on glass slides with VectaShield mounting medium (Vector Laboratories). Sections were imaged using a Leica SP8 confocal microscope.

### Tract-tracing with BDA and biocytin

BDA (molecular weight 3000) or biocytin was injected in vivo into the pallium of two species of cichlids of both sexes, *N. brichardi* (*n* = 5; standard length: 30–49 mm), *A. nigrofasciata* (*n* = 5; standard length: 45–55 mm) and zebrafish *D. rerio* (*n* = 2; standard length: 30 and 35 mm). Fish were anesthetized by immersion in water containing 150–180 mg/L MS222 and set in a device for physical restraint. Water containing 70–80 mg/L MS222 was perfused through the gill for aeration and to maintain the anesthetic condition. A dorsal portion of the cranium was opened to expose the brain. For BDA injections, a glass microelectrode (tip diameters: 12–16 µm) filled with 0.75% BDA solution in 0.05 M Tris-HCl-buffered saline (TBS; pH 7.4) was driven into the pallium with a manipulator (MN-3; Narishige). BDA was injected iontophoretically with square current pulses (+5 µA, 0.5 Hz, 50% duty cycle) passed through the electrodes at three to six places of the pallium each for 5 min with a stimulator (SEN-3301; Nihon Kohden, Japan). For biocytin injections, crystals of biocytin were inserted with a minute insect pin into three to six places of the pallium. After the injection, the cranial opening was closed with either plastic wrap (small fish) or dental cement (Ostron II; GC Dental Products, Japan). Postoperative fish were maintained in aquaria for 21–30 h. The fish were then deeply anesthetized with MS222 (over 200 mg/L) and perfused through the heart with 2% PFA and 1% glutaraldehyde in 0.1 M phosphate buffer (PB), pH 7.4. The brains were removed from the skull and post-fixed in the same fixative at 4 °C for 6–8 h.

The fixed brains were cryo-protected by immersion in 0.1 M PB containing 20% sucrose at 4 °C. Cryo-protected brains were embedded in 5% agarose (type IX, ultra-low gelling temperature) containing 20% sucrose and frozen in n-hexane at −60 °C. Then, frontal sections were cut at a thickness of 40 µm on a cryostat and mounted on gelatin-coated glass slides. The sections were dried and washed once with 0.05 M TBS containing 0.1% Tween 20 (TBST) and twice with TBS. To quench non-specific peroxidase activities, sections were steeped in methanol containing 0.3% H_2_O_2_ and washed three times with TBS and once with 0.03% TBST. Sections were then incubated with a solution of avidin-biotin-peroxidase complex (1:100; VECTASTAIN Elite ABC Standard Kit, Vector Laboratories) overnight. Afterwards, sections were incubated for one hour with 0.05% 3,3’-diaminobenzidine solution in 0.1 M PB containing 0.04% nickel ammonium sulfate and 0.01% H_2_O_2_. The reaction was stopped by washing four times with TBS, and the sections were counterstained with 0.05–0.1% cresyl violet, dehydrated, and coverslipped with Permount (Fisher Scientific).

### Statistics and reproducibility

To determine whether brain mass, body mass, and total number of cells in the brain are correlated in teleosts, a nonparametric Spearman rank correlation test was used on log-transformed data. Previously published data on birds^8^ and mammals^38^ were used for comparison. If a *P* < 0.05 value was found, reduced major axis (RMA) regressions were calculated using the SMATR package^79^ in RStudio v.1.2.5033 and fitted RMA regression lines were added to the plots (Fig. 2). To compare scaling among taxonomic groups, an analysis of covariance (ANCOVA) with post-hoc Sidak corrected pairwise comparisons was used to check for significant differences in the slopes of the regression lines. In groups for which the slopes were statistically homogeneous, the regression lines were compared based on the differences in their intercepts. Body mass and Brain mass, Total number of cells in the brain and Brain mass, and Body mass and Total number of cells in the brain were significantly correlated in all groups (Spearman r ranging from 0.945 to 1; *p* < 0.0001 in all cases). Data on Columbiformes and Galliformes^8^ was plotted as illustration but wasn’t included in the statistical analysis due to the small sample size.

Regression lines for Body mass and Brain mass (Fig. 2a) had significantly different slopes (ANCOVA, *p* < 0.0001). Pairwise comparisons found significant differences in the slopes of Glires and Primates (*p* < 0.001), Primates and Psittacopasserae (*p* = 0.0001) and Primates and Teleosts (*p* < 0.0001). ANCOVA revealed significant differences in the intercepts of the regression lines for Brain mass and Body mass for groups with statistically homogenous slopes (*p* < 0.0001). Pairwise comparisons found significant differences in the intercepts of Glires, Teleosts and Psittacopasserae (*p* < 0.0001 in all cases). Regression lines for Body mass and Total number of cells in the brain (Fig. 2b) had significantly different slopes (ANCOVA, *p* < 0.0001). Pairwise comparisons found significant differences in the slopes of Glires and Primates (*p* < 0.001), Primates and Psittacopasserae (*p* < 0.0001) and Primates and Teleosts (*p* < 0.001). ANCOVA revealed significant differences in the intercepts of the regression lines for Body mass and Total number of cells in the brain for groups with statistically homogenous slopes (*p* < 0.0001). Pairwise comparisons found significant differences in the intercepts of Glires, Teleosts and Psittacopasserae (*p* < 0.05 in all cases). Regression lines for Total number of cells in the brain and Brain mass (Fig. 2c) had significantly different slopes (ANCOVA, *p* < 0.0001). Pairwise comparisons found significant differences in the slopes of Glires and Primates (*p* < 0.01) and Primates and Psittacopasserae (*p* < 0.01). ANCOVA revealed significant differences in the intercepts of the regression lines (*p* < 0.0001). Pairwise comparisons found significant differences in the intercepts of the regression lines for Glires, Teleosts, Primates and Psittacopasserae (*p* < 0.0001 in all cases), with the exception of the intercepts of Glires and Primates (*p* = 0.08). In order to determine the degree of encephalization of the teleost species sampled in this study, a phylogenetically corrected brain-body allometric slope was estimated using PGLS at the Class level on species means of log_10_ brain and log_10_ body mass data of the species sampled in this study along with previously published actynopterygian data by Tsuboi et al. ^5^ using RStudio v.1.2.5033 with the CAPER package v.1.0.1 (Fig. 3). Residual variance was modeled according to Brownian motion^80^ and phylogenetic signal was estimated using Pagel’s λ^81^. Phylogenetic relationships between teleost species were based on previously published phylogenetic trees^82^. The phylogenetic regression slope for actinopterygians was of 0.50 ± 0.01 (Adjusted R^2^: 0.8382, *t* = 65.978, *p* < 0.0001). Encephalization was then determined by extracting the residuals of log_10_-log_10_ brain and body mass for each species of the dataset to remove allometry in brain size^83^. The 11 species studied were ranked based on the value of their residual (Supplementary Table 1).

To determine whether there exists a correlation between the degree of encephalization and relative mass and relative number of cells (expressed as the percentage of total brain mass and percentage of total brain cells, respectively) of the five major brain structures dissected, a nonparametric Spearman rank correlation test was used, as there was no way to ascertain the normal distribution of these data. We arranged species by decreasing order of encephalization (Supplementary Fig. 4). The test was performed in GraphPad Prism v.9.0.0 (GraphPad Software) on species means. A significant negative correlation was found between encephalization and the relative mass and relative number of cells in the rHind (Supplementary Fig. 4e, Spearman r: −0.7091, *p* = 0.0268 and Spearman r: −0.6727, *p* = 0.039, respectively). No significant correlation with encephalization was found in the four other brain structures for either relative mass or relative number of cells (Supplementary Fig. 4a–d, Tel relative mass: Spearman r: 0.4788, *p* = 0.1663; relative number of cells: Spearman r: 0.01818, *p* = 0.973; TeO relative mass: Spearman r: −0.1394, *p* = 0.7072; relative number of cells: Spearman r: 0.1394, *p* = 0.7072; rForeMid relative mass: Spearman r: 0.3333, *p* = 0.3487; relative number of cells: Spearman r: −0.4788, *p* = 0.1663; Cb relative mass: Spearman r: −0.1273, *p* = 0.7330; relative number of cells: Spearman r: 0.4545, *p* = 0.1912).

In order to compare species to species the relative mass, absolute and relative number of cells in major brain structures, normality of the data was tested using Shapiro-Wilk’s test. As normality was not verified for all the species studied, and considering the small sample size, nonparametric Kruskal-Wallis and Dunn’s post hoc tests were used to assess the inter-species differences in relative mass, absolute and relative number of cells in the five dissected brain structures. All tests were performed in GraphPad Prism v. 9.0.0. Significant differences were found in the absolute number of cells in all five structures (Kruskal-Wallis, *p* < 0.001 in all cases). However, post-hoc pairwise comparisons revealed significant differences that were inconsistent across species and brain structures, the only consistently found difference across all structures being between *D. rerio* and *C. anchorago* (Dunn’s test, *p* < 0.05 in all cases). Significant differences were found in the relative number of cells in all five structures (Kruskal-Wallis, *p* < 0.05 in all cases). However, post-hoc pairwise comparisons revealed significant differences that were inconsistent across species and brain structures. Significant differences were found in the relative mass in all five structures (Kruskal-Wallis, *p* < 0.05 in all cases). However, post-hoc pairwise comparisons didn’t reveal significant differences between species across the five structures, except for a modest difference in the relative mass of the rHind between *A. mexicanus*, *C. anchorago* and *T. hardwicke* (Dunn’s test, *p* = 0.0307 and *p* = 0.0317, respectively), and in the relative mass of the Tel between *C. anchorago* and *S. trutta* (Dunn’s test, *p* = 0.0254).

Among the teleost species studied, wrasses display the most complex behavioral phenotypes. To determine whether this behavioral repertoire is associated with differences in relative mass and relative number of cells in the five major brain structures compared to other teleosts, the three species of wrasse (*C. anchorago* (*n* = 4), *T. hardwicke* (*n* = 3) and *L. dimidiatus* (*n* = 3)) were grouped together (*n* = 10) and compared to all the other species (*M. zebra* (*n* = 3), *N. brichardi* (*n* = 5), *O. boops* (*n* = 3), *A. nigrofasciata* (*n* = 5), *A. mexicanus* (*n* = 5), *D. rerio* (*n* = 5) and *S. trutta* (*n* = 4), grouped as “other fish” (*n* = 30)) (Fig. 5). Regarding the relative mass, wrasses had a significantly larger Tel (Fig. 5a) and rForeMid (Fig. 5c) compared to other teleosts (Mann-Whitney’s test, *p* < 0.0001 and *p* = 0.0031, respectively), and a significantly smaller Cb (Fig. 5d) and rHind (Fig. 5e) (*p* = 0.0011 and *p* < 0.0001, respectively). No significant differences were found in the relative mass of the TeO (Fig. 5b, *p* = 0.5483). Regarding the relative number of cells, Wrasses had a significantly lower relative number of cells in the rHind (Fig. 5j) compared to the other teleosts (Mann-Whitney’s test, *p* < 0.0001). No significant differences were found in the relative number of cells of the other four structures (Fig. 5f–i, Tel: *p* = 0.0538; TeO: *p* = 0.3626; rForeMid: *p* = 0.1983; Cb: *p* = 0.8658). Normality and Mann-Whitney tests were performed in GraphPad Prism v. 9.0.0.

Another analysis was done in order to assess the differences in relative mass and relative number of cells in the brain structures: as cichlids appeared to have a large rForeMid, we decided to compare them to wrasses and to the other species of teleosts studied here (Supplementary Fig. 6). *A. mexicanus* (*n* = 5), *D. rerio* (*n* = 5) and *S. trutta* (*n* = 4), were grouped together as an “outgroup” (*n* = 14). In both cases, as normality could not be satisfied for all structures in all groups, a nonparametric Kruskal-Wallis test was used. All tests were performed in GraphPad Prism v. 9.0.0. Regarding the relative mass, wrasses and cichlids had a significantly larger Tel (Supplementary Fig. 6a) and rForeMid (Supplementary Fig. 6c) compared to the “outgroup” (Kruskal-Wallis test, *p* < 0.01 in both structures), and a significantly smaller TeO (Supplementary Fig. 6b) (*p* < 0.05 in all cases). Additionally, wrasses had a larger Tel compared to cichlids (Supplementary Fig. 6a) (*p* = 0.0324). Regarding the relative number of cells, wrasses had a significantly lower relative number of cells in the rHind (Supplementary Fig. 6j) while cichlids had a significantly higher relative number of cells in the rForeMid (Supplementary Fig. 6h) compared to the “outgroup” (Kruskal-Wallis test, *p* < 0.0001 and *p* = 0.0137, respectively). No significant differences were found in the relative number of cells in the rForeMid between wrasses and cichlids (*p* = 0.0506).

### Reporting summary

Further information on research design is available in the Nature Portfolio Reporting Summary linked to this article.

### Supplementary information

Supplementary Information

Description of Additional Supplementary Files

Supplementary Movies 1

Supplementary Movies 2

Supplementary Movies 3

Supplementary Movies 4

Supplementary Movies 5

Supplementary Data 1

Reporting Summary

## Data Availability

Data reported in this paper is accessible in Supplementary Data 1. This paper does not report original code.
