# Supplementary material for: Different ways of evolving tool-using brains in teleosts and amniotes
Source: Commun Biol. 2024 Jan 12;7:88. doi: 10.1038/s42003-023-05663-8 (PMC10786859; doi:10.1038/s42003-023-05663-8)
Supplement: Supplementary file 1 — Supplementary Information [file 42003_2023_5663_MOESM1_ESM.pdf]

SUPPLEMENTARY INFORMATION

SUPPLEMENTARY MOVIES TITLES

Supplementary Movie 1

3D selective visualization of inferior lobe fiber tracts in the wrasse (*Choerodon anchorago*) brain.

Supplementary Movie 2

3D selective visualization of inferior lobe fiber tracts in the trout (*Salmo trutta*) brain.

Supplementary Movie 3

3D selective visualization of inferior lobe fiber tracts in the *Astyanax* surface fish (*Astyanax mexicanus*) brain.

Supplementary Movie 4

3D selective visualization of inferior lobe fiber tracts in the zebrafish (*Danio rerio*) brain.

Supplementary Movie 5

3D selective visualization of inferior lobe fiber tracts in the cichlid (*Neolamprologus brichardi*) brain.

Supplementary Table 1. Residuals obtained by fitting a $\log_{10}$-$\log_{10}$ regression of brain mass against body mass for 11 species of teleost. All values are rounded to the nearest ten thousandths.

Species	Residual	Residual excluding the large *C. anchorago* individual
Thalassoma hardwicke	0.3797	0.3792
Choerodon anchorago	0.3420	0.4879
Opthalmotilapia boops	0.2795	0.2790
Labroides dimidiatus	0.2458	0.2451
Salmo trutta	0.2171	0.2170
Maylandia zebra	0.2012	0.2008
Astyanax mexicanus	0.1549	0.1543
Oryzias latipes	0.1530	0.1520
Neolamprologus brichardi	0.0708	0.0703
Amatitlania nigrofasciata	0.0179	0.0176
Danio rerio	-0.1409	-0.1417

Supplementary File 1: **Statistical analysis excluding the large individual of *Choerodon anchorago***

Wrasses in this study were wild caught and tended to be young adults, but one large adult of *Choerodon anchorago* weighing around ten times as much as the other individuals was also sampled. This individual weighed 297,54 g and had a brain mass of 648,9 mg. As this individual diverged from the other 3 *C. anchorago* individuals sampled, we verified whether excluding it from our statistical analysis changed any of our conclusions. The results of this analysis are detailed below.

<u>Cellular scaling rules of teleost brains</u>

To determine whether brain mass, body mass, and total number of cells in the brain are correlated in Teleosts, a nonparametric Spearman rank correlation test was used on $\log_{10}$-transformed data. Previously published data on birds[1] and mammals[2] were used for comparison. If a $P<0.05$ value was found, reduced major axis (RMA) regressions were calculated using the SMATR package[3] in RStudio v.1.2.5033 and fitted RMA regression lines were added to the plots (Supplementary Fig. 1a-c). To compare scaling among taxonomic groups, an analysis of covariance (ANCOVA) with post-hoc Sidak corrected pairwise comparisons was used to check for significant differences in the slopes of the regression lines. In groups for which the slopes were statistically homogeneous, the regression lines were compared based on the differences in their intercepts.

Body mass and Brain mass, Total number of cells in the brain and Brain mass, and Body mass and Total number of cells in the brain were significantly correlated in all groups (Spearman r ranging from 0.945 to 1; $p<0.0001$ in all cases). Data on

Columbiformes and Galliformes[1] was plotted as illustration but wasn't included in the statistical analysis due to the small sample size.

Regression lines for Body mass and Brain mass (Supplementary Fig. 1a) had significantly different slopes (ANCOVA, $p<0.0001$). Pairwise comparisons found significant differences in the slopes of Glires and Primates ($p<0.001$), Primates and Psittacopasserae ($p=0.0001$) and Primates and Teleosts ($p<0.0001$). ANCOVA revealed significant differences in the intercepts of the regression lines for Brain mass and Body mass for groups with statistically homogenous slopes ($p<0.0001$). Pairwise comparisons found significant differences in the intercepts of Glires, Teleosts and Psittacopasserae ($p<0.0001$ in all cases).

Regression lines for Body mass and Total number of cells in the brain (Supplementary Fig. 1b) had significantly different slopes (ANCOVA, $p<0.0001$). Pairwise comparisons found significant differences in the slopes of Glires and Primates ($p<0.001$), Primates and Psittacopasserae ($p<0.0001$) and Primates and Teleosts ($p<0.001$). ANCOVA revealed significant differences in the intercepts of the regression lines for Body mass and Total number of cells in the brain for groups with statistically homogenous slopes ($p<0.0001$). Pairwise comparisons found significant differences in the intercepts of Glires, Teleosts and Psittacopasserae ($p<0.05$ in all cases).

Regression lines for Total number of cells in the brain and Brain mass (Supplementary Fig. 1c) had significantly different slopes (ANCOVA, $p<0.0001$). Pairwise comparisons found significant differences in the slopes of Glires and Primates ($p<0.01$) and Primates and Psittacopasserae ($p<0.01$). ANCOVA revealed significant differences in the intercepts of the regression lines ($p<0.0001$). Pairwise comparisons found significant differences in the intercepts of the regression lines for Glires,

Teleosts, Primates and Psittacopasserae ($p<0.0001$ in all cases), with the exception of the intercepts of Glires and Primates ($p=0.08$).

Overall, the statistical significance of the results was unchanged when the large *C. anchorago* individual was excluded from the dataset.

<u>Degree of encephalization of sampled species</u>

In order to determine the degree of encephalization of the teleost species sampled in this study, a phylogenetically corrected brain-body allometric slope was estimated using phylogenetically generalized least squares regression test (PGLS) at the Class level on species means of $\log_{10}$ brain and $\log_{10}$ body mass data of the species sampled in this study along with previously published actynopterygians data by Tsuboi et al[4]. using RStudio v.1.2.5033 with the CAPER package v.1.0.1 (Supplementary Fig. 1d). Residual variance was modelled according to Brownian motion[5] and phylogenetic signal was estimated using Pagel's λ[6]. Phylogenetic relationships between teleost species were based on previously published phylogenetic trees[7]. The phylogenetic regression slope for actinopterygians was of 0.50 ± 0.01 (Adjusted R^2: 0.8379, $t=65.891$, $p<0.0001$).

Encephalization was then determined by extracting the residuals of $\log_{10}$-$\log_{10}$ brain and body mass for each species of the dataset to remove allometry in brain size[8]. The 11 species studied were ranked based on the value of their residual (Supplementary Table 1). The exclusion of the large *C. anchorago* individual gave a residual of 0.488 for *C. anchorago*, placing it above the wrasse *Thalassoma hardwicke*.

<u>Encephalization and relative mass and relative number of cells of major brain structures</u>

To determine whether there exists a correlation between the degree of encephalization and relative mass and relative number of cells (expressed as the percentage of total brain mass and percentage of total brain cells, respectively) of the five major brain structures dissected, a nonparametric Spearman rank correlation test was used, as there was no way to ascertain the normal distribution of these data. We arranged species by decreasing order of encephalization (Supplementary Fig. 2). The test was performed in GraphPad Prism v.9.0.0 (GraphPad Software, San Diego, CA, USA) on species means. A significant negative correlation was found between encephalization and the relative mass and relative number of cells in the rHind (Supplementary Fig. 2e, Spearman r: -0.6970, p=0.0306 and Spearman r: -0.6606, p=0.0438, respectively). No significant correlation with encephalization was found in the four other brain structures for either relative mass or relative number of cells (Supplementary Figs. 2a-d, Tel relative mass: Spearman r: 0.5152, p=0.1334; relative number of cells: Spearman r: -0.01818, p=0.973; TeO relative mass: Spearman r: -0.2242, p=0.5367; relative number of cells: Spearman r: 0.09091, p=0.8113; rForeMid relative mass: Spearman r: 0.3818, p=0.2788; relative number of cells: Spearman r: -0.2242, p=0.5367; Cb relative mass: Spearman r: -0.1758, p=0.6321; relative number of cells: Spearman r: 0.3212, p=0.3679).

Overall, the statistical significance of the results was unchanged when the large *C. anchorago* individual was excluded from the dataset.

<u>Species to species comparison of relative mass, absolute and relative number of cells in major brain structures</u>

Normality of the data was tested using Shapiro-Wilk's test. As normality was not verified for all the species studied, and considering the small sample size,

nonparametric Kruskal-Wallis and Dunn’s post hoc tests were used to assess the inter-species differences in relative mass, absolute and relative number of cells in the five dissected brain structures. All tests were performed in GraphPad Prism v. 9.0.0.

Significant differences were found in the absolute number of cells in all five structures (Kruskal-Wallis, $p<0.001$ in all cases). However, post-hoc pairwise comparisons revealed significant differences that were inconsistent across species and brain structures, the only consistently found difference across all structures being between *D. rerio* and *C. anchorago* (Dunn’s test, $p<0.05$ in all cases).

Significant differences were found in the relative number of cells in all five structures (Kruskal-Wallis, $p<0.05$ in all cases). However, post-hoc pairwise comparisons revealed significant differences that were inconsistent across species and brain structures.

Significant differences were found in the relative mass of the Tel, TeO, rForeMid and rHind (Kruskal-Wallis, $p<0.01$ in all cases). No significant difference was found between species in the relative mass of the Cb (Kruskal-Wallis, $p=0.0649$). However, post-hoc pairwise comparisons didn’t reveal significant differences between species across the four structures, except for a modest difference in the relative mass of the rHind between *A. mexicanus* and *T. hardwicke* (Dunn’s test, $p=0.0292$).

Overall, the statistical significance of the results was unchanged when the large *C. anchorago* individual was excluded from the dataset.

<u>Comparison of relative mass and relative number of cells in major brain structures based on behavioral repertoire</u>

Among the teleost species studied, wrasses display the most complex behavioral phenotypes. To determine whether this behavioral repertoire is associated with

differences in relative mass and relative number of cells in major brain structures compared to other teleosts, the three species of wrasse (*C. anchorago* (n=3), *T. hardwicke* (n=3) and *L. dimidiatus* (n=3)) were grouped together (n=9) and compared to all the other species (*M. zebra* (n=3), *N. brichardi* (n=5), *O. boops* (n=3), *A. nigrofasciata* (n=5), *A. mexicanus* (n=5), *D. rerio* (n=5) and *S. trutta* (n=4), grouped as "Other fish" (n=30)). As normality could not be satisfied for all structures in both groups, a nonparametric Mann-Whitney test was used. Regarding the relative mass, wrasses had a significantly larger Tel and rForeMid compared to other teleosts (Mann-Whitney's test, $p<0.0001$ and $p=0.008$, respectively), and a significantly smaller Cb and rHind ($p=0.0035$ and $p<0.0001$, respectively). No significant differences were found in the relative mass of the TeO ($p=0.8571$). Regarding the relative number of cells, wrasses had a significantly lower relative number of cells in the rHind compared to the other teleosts (Mann-Whitney's test, $p<0.0001$). No significant differences were found in the relative number of cells of the other four structures (Tel: $p=0.1226$; TeO: $p=0.1494$; rForeMid: $p=0.0636$; Cb: $p=0.9088$). Normality and Mann-Whitney tests were performed in GraphPad Prism v. 9.0.0.

Overall, the statistical significance of the results was unchanged when the large *C. anchorago* individual was excluded from the dataset.

<u>Comparison of relative mass and relative number of cells in major brains structures of wrasses and cichlids compared to the outgroup</u>

As cichlids appeared to have a large rForeMid, we decided to compare them to wrasses and to the other species of teleosts studied here. Species were grouped into different clusters: *M. zebra* (n=3), *N. brichardi* (n=5), *O. boops* (n=3) and *A. nigrofasciata* (n=5), all members of the Cichlidae family, were grouped as "cichlids"

(n=16). *C. anchorago* (n=3), *T. hardwicke* (n=3) and *L. dimidiatus* (n=3), members of the Labridae family, were grouped as “wrasses” (n=9). *A. mexicanus* (n=5) (Characidae), *D. rerio* (n=5) (Cyprinidae) and *S. trutta* (n=4) (Salmonidae) being phylogenetically distant, were grouped together as an “outgroup” (n=14) because of the n=1 species per family sample size.

These groups were compared to one another using nonparametric Kruskal-Wallis tests, as normality could not be satisfied for all structures in all groups (Supplementary Fig. 3). Regarding the relative mass, wrasses and cichlids had a significantly larger Tel (Supplementary Fig. 3a) and rForeMid (Supplementary Fig. 3c) compared to the "outgroup" (Kruskal-Wallis test, $p<0.005$ in both structures). Cichlids also had a significantly smaller TeO (Supplementary Fig. 3b) compared to the outgroup, and a larger rHind compared to wrasses (Supplementary Fig. 3e) ($p<0.0001$ and $p=0.0050$, respectively).

Regarding the relative number of cells, wrasses had a significantly lower relative number of cells in the rHind compared to cichlids and the "outgroup" (Supplementary Fig. 3j) (Kruskal-Wallis test, $p=0.0036$ and $p<0.0001$, respectively), while cichlids had a significantly higher relative number of cells in the rForeMid (Supplementary Fig. 3h) compared to both the "outgroup" and wrasses ($p=0.0114$ and $p=0.0116$, respectively). Thus, excluding the large individual of *C. anchorago* from the dataset led to the relative number of cells in the rForeMid of cichlids to become significantly higher compared to wrasses. No significant differences were found in the relative number of cells of the other three structures. All tests were performed in GraphPad Prism v. 9.0.0.

SUPPLEMENTARY REFERENCES

1. Olkowicz, S., Kocourek, M., Lučan, R.K., Porteš, M., Fitch, W.T., Herculano-Houzel, S., and Němec, P. (2016). Birds have primate-like numbers of neurons in the forebrain. Proc. Natl. Acad. Sci. *113*, 7255–7260. 10.1080/19419899.2013.835743.

2. Herculano-Houzel, S., Catania, K., Manger, P.R., and Kaas, J.H. (2015). Mammalian Brains Are Made of These: A Dataset of the Numbers and Densities of Neuronal and Nonneuronal Cells in the Brain of Glires, Primates, Scandentia, Eulipotyphlans, Afrotherians and Artiodactyls, and Their Relationship with Body Mass. Brain. Behav. Evol. *86*, 145–163. 10.1159/000437413.

3. Warton, D.I., Duursma, R.A., Falster, D.S., and Taskinen, S. (2012). smatr 3– an R package for estimation and inference about allometric lines. Methods Ecol. Evol. *3*, 257–259. 10.1111/j.2041-210X.2011.00153.x.

4. Tsuboi, M., van der Bijl, W., Kopperud, B.T., Erritzøe, J., Voje, K.L., Kotrschal, A., Yopak, K.E., Collin, S.P., Iwaniuk, A.N., and Kolm, N. (2018). Breakdown of brain–body allometry and the encephalization of birds and mammals. Nat. Ecol. Evol. *2*, 1492–1500. 10.1038/s41559-018-0632-1.

5. Felsenstein, J. (1985). Phylogenies and the Comparative Method. Am. Nat. *125*, 1–15.

6. Lynch, M. (1991). Methods for the analysis of comparative data in evolutionary biology. Evol. Int. J. Org. Evol. *45*, 1065–1080. 10.1111/j.1558-5646.1991.tb04375.x.

7. Rabosky, D.L., Santini, F., Eastman, J., Smith, S.A., Sidlauskas, B., Chang, J., and Alfaro, M.E. (2013). Rates of speciation and morphological evolution are correlated across the largest vertebrate radiation. Nat. Commun. *4*, 1958. 10.1038/ncomms2958.

8. Sol, D., Sayol, F., Ducatez, S., and Lefebvre, L. (2016). The life-history basis of behavioural innovations. Philos. Trans. R. Soc. Lond. B. Biol. Sci. *371*, 20150187. 10.1098/rstb.2015.0187.

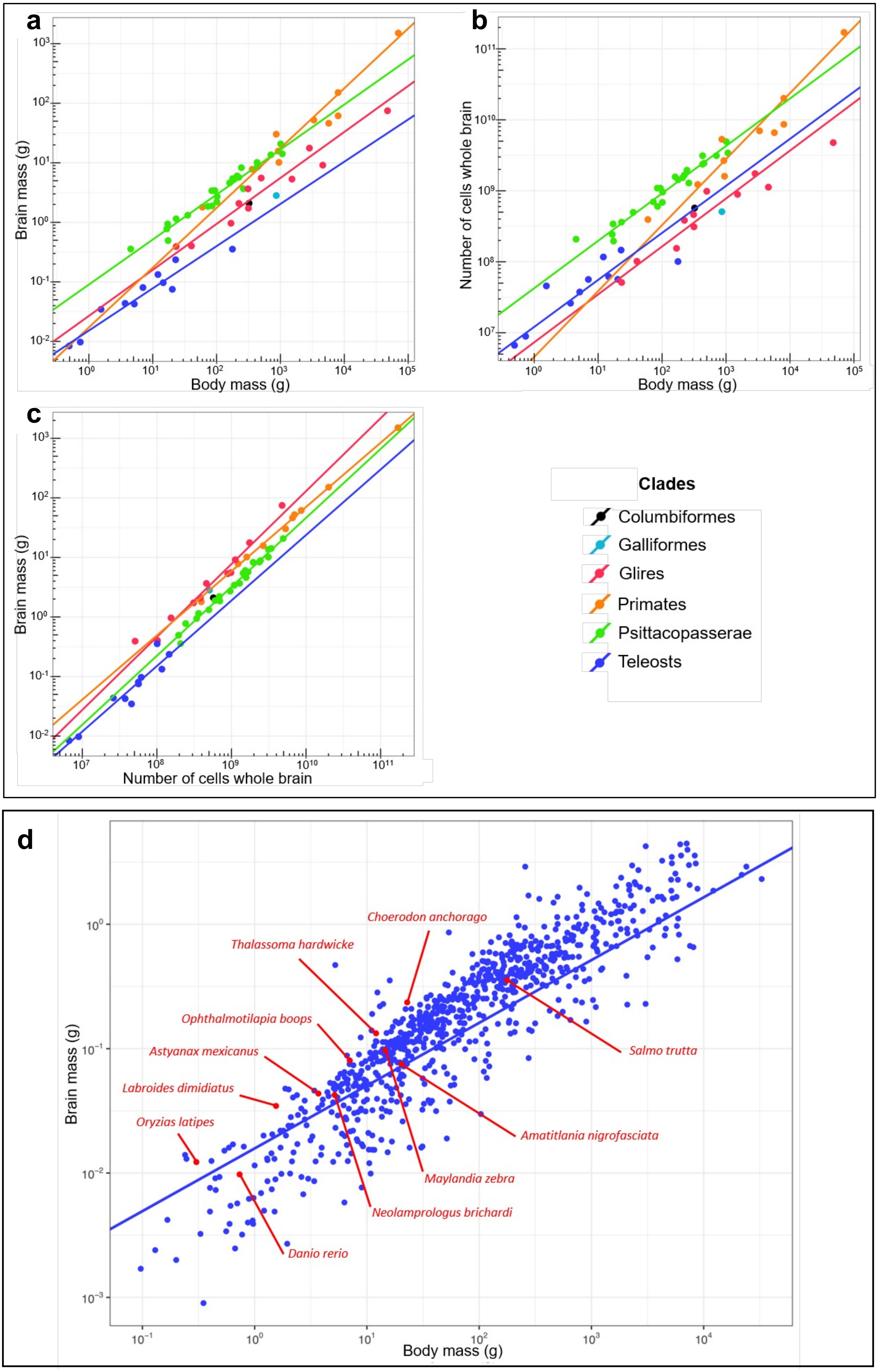

Supplementary Fig. 1. Teleosts have small, cell-dense brains that contain more cells than the brains of rodents of similar body mass even after removal of the large *Choerodon anchorago* individual from the analysis.

(a-c) The fitted reduced major axis (RMA) regression lines are displayed only for correlations that are significant. Each point represents the mean value of a species. X and y axes are in $\log_{10}$ scales. All regression lines are significantly different, except for the regression lines of Glires and Primates in plot (c).

(a) Brain mass plotted as a function of body mass. Teleosts have smaller brains than birds and mammals of similar body mass.

(b) Total number of cells in the brain plotted as a function of body mass. Teleost brains contain less cells than bird and primate brains, but more cells than the brains of rodents of similar body mass.

(c) Brain mass plotted as a function of total number of cells in the brain. Cellular density inside the teleost brain is higher than in birds and mammals.

See also Supplementary Table 1. For statistics, see Supplementary File 1.

(d) Encephalization in 11 species of teleosts compared to a large dataset of actinopterygians after removal of the large *Choerodon anchorago* individual. Brain mass is plotted as a function of body mass, and the phylogenetically corrected (phylogenetically generalized least squares regression test, PGLS) allometric line is shown. Each point represents the mean value of a species. X and y axes are in $\log_{10}$ scales. The phylogenetic regression slope for actinopterygians is of 0.50 ± 0.01. Adjusted R^2: 0.8379, t=65.891, $p<0.0001$.

See also Supplementary Table 1 and Supplementary File 1.

Glires = rodents and lagomorphs. Psittacopasserae = Passeriformes (songbirds) and Psittaciformes (parrots).

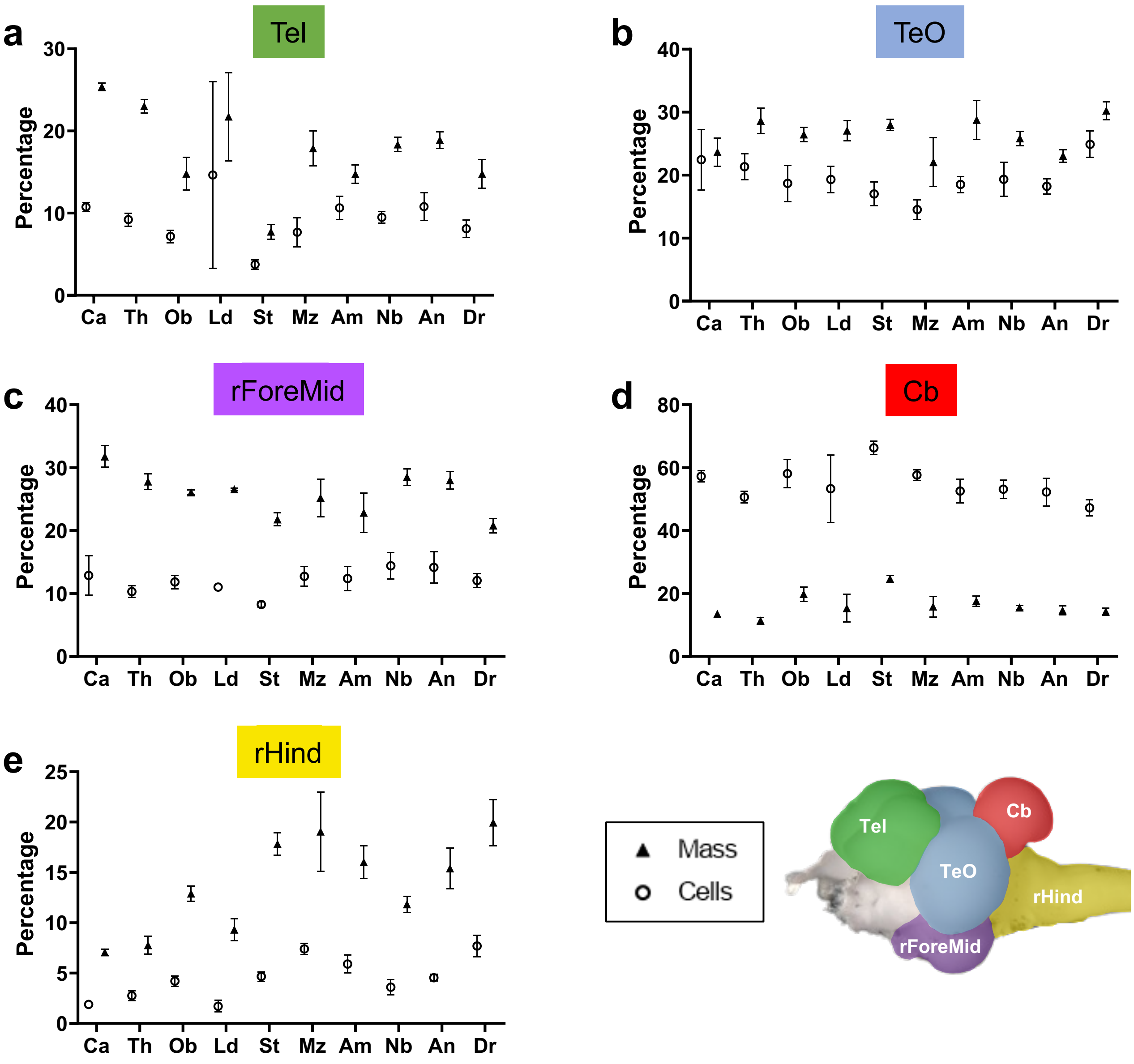

Supplementary Fig. 2. Encephalization in teleosts is not correlated with an increase in the relative mass or relative number of cells in the telencephalon (Tel) even after removal of the large *Choerodon anchorago* individual from the analysis.

(a-e) Relative mass (triangles) and relative number of cells (open circles) of the Tel (a), TeO (b), rForeMid (c), Cb (d), and rHind (e) of ten species of teleosts. Species are ranked from most encephalized (left) to least encephalized (right). Each point represents the mean value of a species. Error bars show mean ± SD. Spearman's rank correlation test was used. Relative mass and relative number of cells in the rH (e) are negatively correlated with encephalization (Spearman r: -0.697, p=0.031 and Spearman r: -0.661, p=0.044), whereas no significant correlation exists for the other regions.

Species: Am: *Astyanax mexicanus*; An: *Amatitlania nigrofasciata*; Ca: *Choerodon anchorago*; Dr: *Danio rerio*; Ld: *Labroides dimidiatus*; Mz: *Maylandia zebra*; Nb: *Neolamprologus brichardi*; Ob: *Ophtalmotilapia boops*; St: *Salmo trutta*; Th: *Thalassoma hardwicke*.

Brain regions: Cb: cerebellum, rForeMid: rest of the forebrain/midbrain; rHind: rest of the hindrain; Tel: telencephalon; TeO: optic tectum.

See also Supplementary File 1.

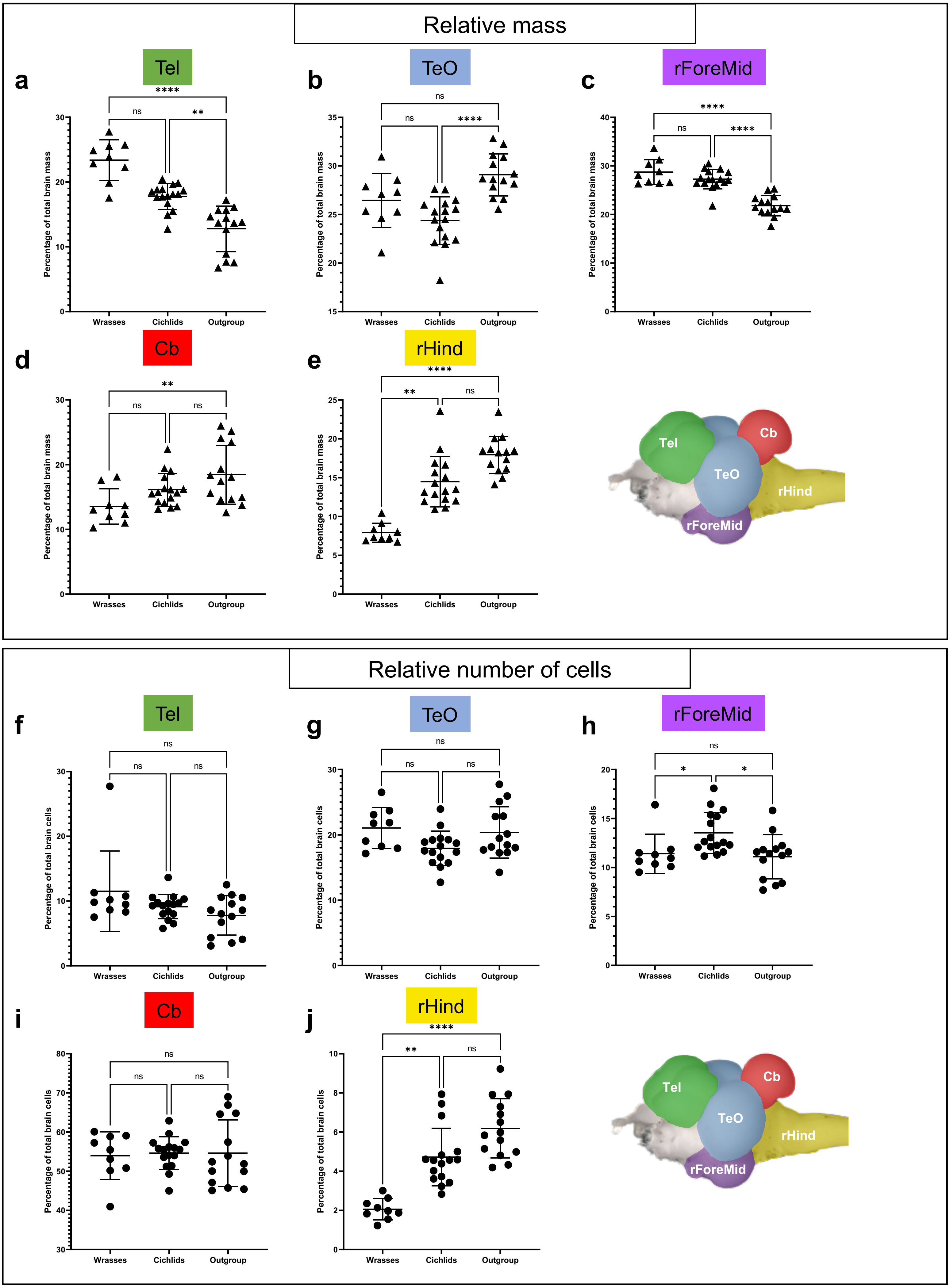

Supplementary Fig. 3. Wrasses and cichlids have a relatively larger telencephalon (Tel) and rest of the forebrain/midbrain (rForeMid) compared to other teleosts even after removal of the large *Choerodon anchorago* individual from the analysis.

Four species of cichlids (*Amatitlania nigrofasciata, Maylandia zebra, Neolamprologus brichardi, Ophtalmotilapia boops*), and three species of wrasses (*Choerodon anchorago*, *Labroides dimidiatus*, *Thalassoma hardwicke*) were compared with three species of teleosts of various orders («Outgroup»: *Astyanax* mexicanus, *Danio rerio, Salmo* trutta).
(a-e) Comparison of the relative mass of the Tel (a), TeO (b), rForeMid (c), Cb (d), and rHind (e). Wrasses and cichlids have a relatively larger Tel and rForeMid compared to the outgroup.
(f-j) Comparison of the relative number of cells in the Tel (f), TeO (g), rForeMid (h), Cb (i), and rHind (j). When removing the large individual of *Choerodon anchorago*, wrasses and cichlids don't have a larger proportion of cells in the Tel, but cichlids have a larger proportion in the rForeMid compared to the outgroup and to wrasses. Statistical analysis: Kruskal-Wallis test. Each point represents individual values. Error bars: mean ± SD.
ns: non significant, *$p<0.05$, **$p<0.01$, ****$p<0.0001$.
Brain regions: Cb: cerebellum, rForeMid: rest of the forebrain/midbrain; rHind: rest of the hindrain; Tel: telencephalon; TeO: optic tectum.
See also Supplementary File 1.

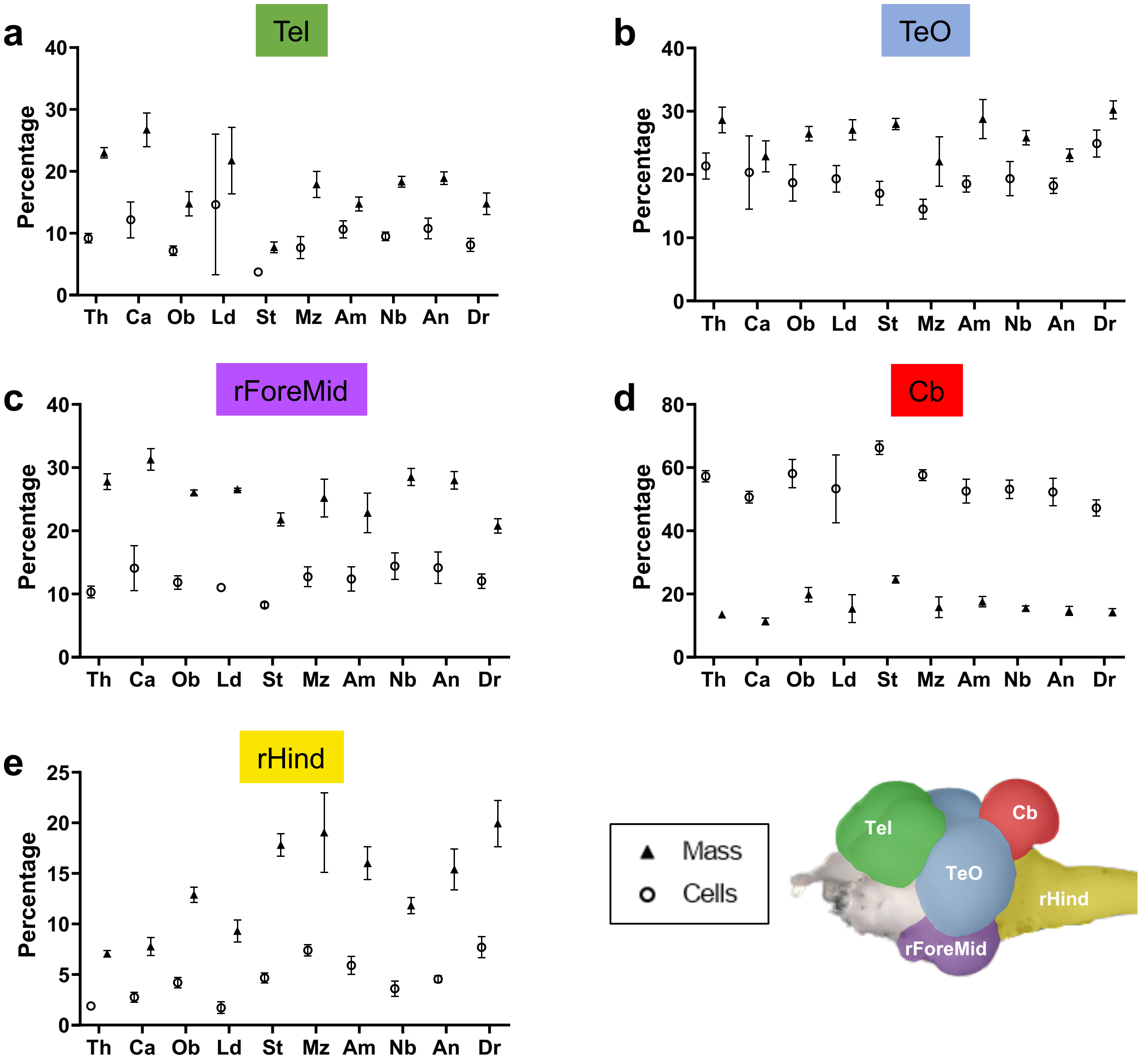

Supplementary Figure 4. Encephalization in teleosts is not correlated with an increase in the relative mass or relative number of cells in the telencephalon (Tel).

(a-e) Relative mass (triangles) and relative number of cells (open circles) of the Tel (a), TeO (b), rForeMid (c), Cb (d), and rHind (e) of ten species of teleosts. Species are ranked from most encephalized (left) to least encephalized (right). Each point represents the mean value of a species. Error bars show mean ± SD. Spearman's rank correlation test was used. Relative mass and relative number of cells in the rHind (e) are negatively correlated with encephalization (Spearman r: -0.709, p=0.027 and Spearman r: -0.673, p=0.039), whereas no significant correlation exists for the other regions.

Species: Am: *Astyanax mexicanus*; An: *Amatitlania nigrofasciata*; Ca: *Choerodon anchorago*; Dr: *Danio rerio*; Ld: *Labroides dimidiatus*; Mz: *Maylandia zebra*; Nb: *Neolamprologus brichardi*; Ob: *Ophtalmotilapia boops*; St: *Salmo trutta*; Th: *Thalassoma hardwicke*.

Brain regions: Cb: cerebellum, rForeMid: rest of the forebrain/midbrain; rHind: rest of the hindrain; Tel: telencephalon; TeO: optic tectum.

See also "Methods" section and Supplementary Methods.

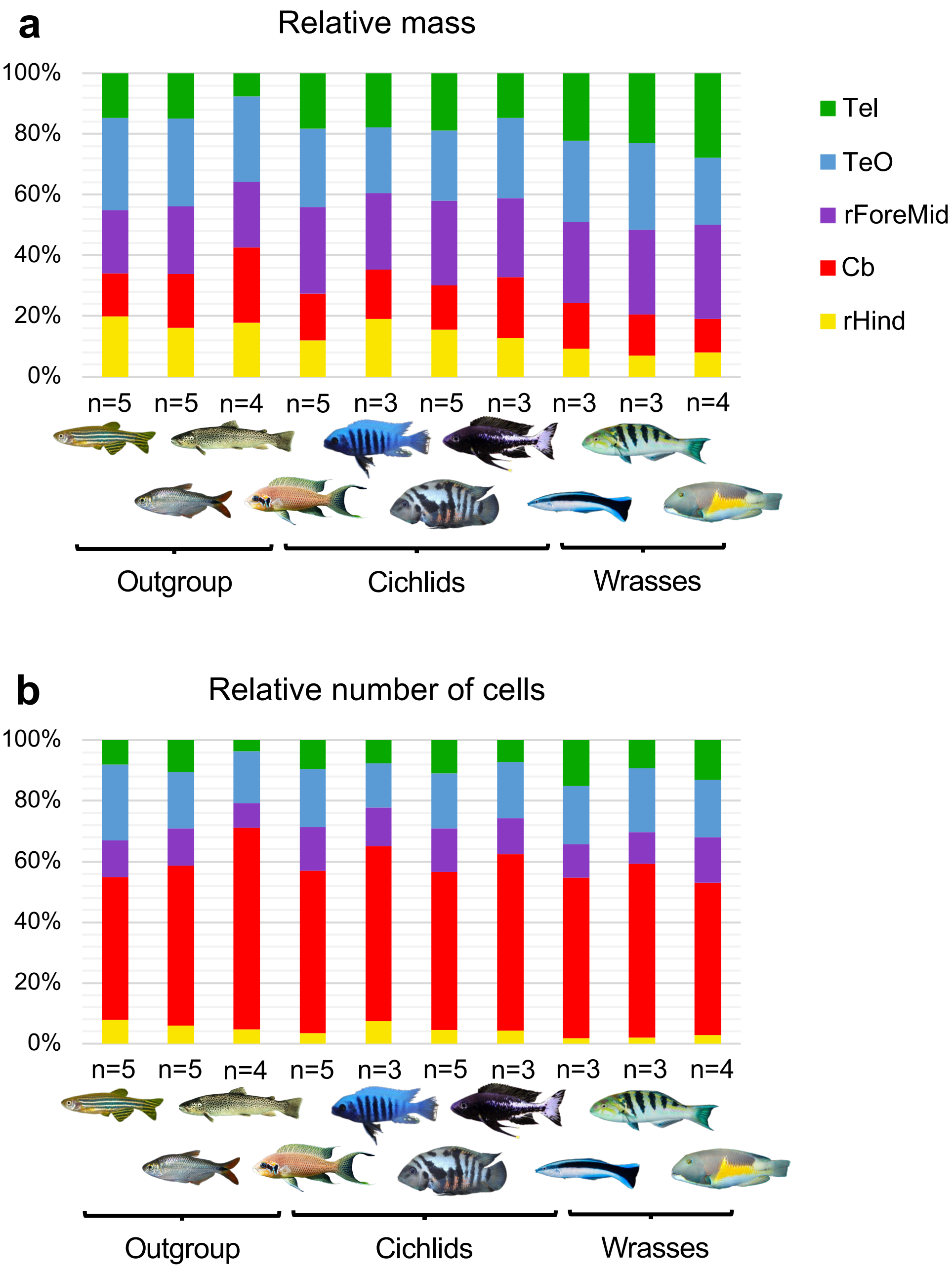

Supplementary Fig. 5. Comparison of relative mass and number of cells in the brains of teleosts.

Mass distribution and cellular composition of teleost brains appear to be similar across phylogeny when species are compared one to one. Relative mass (a) and number of cells (b) of the Tel, TeO, rForeMid, Cb and rHind of ten teleost species. Values are mean percentages per species.

Species from left to right: “outgroup” (*Danio rerio*, *Astyanax mexicanus*, *Salmo trutta*), Cichlids (*Neolamprologus brichardi*, *Maylandia zebra*, *Amatitlania nigrofasciata*, *Ophthalmotilapia boops*), Wrasses (*Labroides dimidiatus*, *Thalassoma hardwicke*, *Choerodon anchorago)*.

Brain regions: Cb: cerebellum, rForeMid: rest of the forebrain/midbrain; rHind: rest of the hindrain; Tel: telencephalon; TeO: optic tectum.

See also “Methods” section.

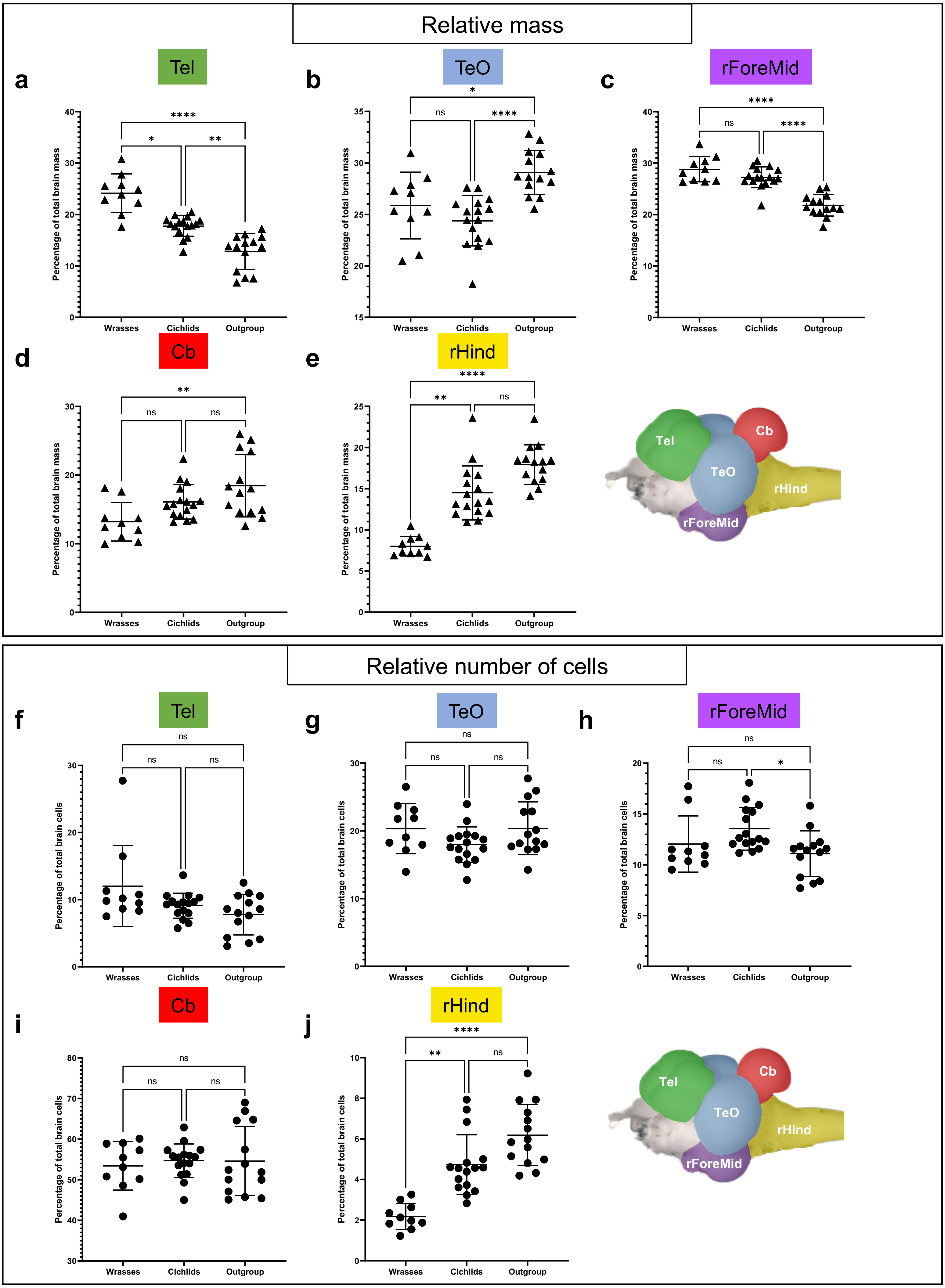

Supplementary Fig. 6. Wrasses and cichlids have a relatively larger telencephalon (Tel) and rest of the forebrain/midbrain (rForeMid) compared to other teleosts.

Four species of cichlids (*Amatitlania nigrofasciata, Maylandia zebra, Neolamprologus brichardi, Ophtalmotilapia boops*), and three species of wrasses (*Choerodon anchorago*, *Labroides dimidiatus*, *Thalassoma hardwicke*) were compared with three species of teleosts of various orders («Outgroup»: *Astyanax* mexicanus, *Danio rerio, Salmo* trutta).
(a-e) Comparison of the relative mass of the Tel (a), TeO (b), rForeMid (c), Cb (d), and rHind (e). Wrasses and cichlids have a relatively larger Tel and rForeMid compared to the outgroup, and wrasses have a relatively larger Tel compared to cichlids. (f-j) Comparison of the relative number of cells in the Tel (f), TeO (g), rForeMid (h), Cb (i), and rHind (j). Wrasses and cichlids don't have a larger proportion of cells in the Tel, but cichlids have a larger proportion in the rForeMid compared to the outgroup. Statistical analysis: Kruskal-Wallis test. Each point represents individual values. Error bars: mean ± SD.
ns: non significant, *p<0.05, **p<0.01, ****p<0.0001.
Brain regions: Cb: cerebellum, rForeMid: rest of the forebrain/midbrain; rHind: rest of the hindrain; Tel: telencephalon; TeO: optic tectum.
See also "Methods" section.

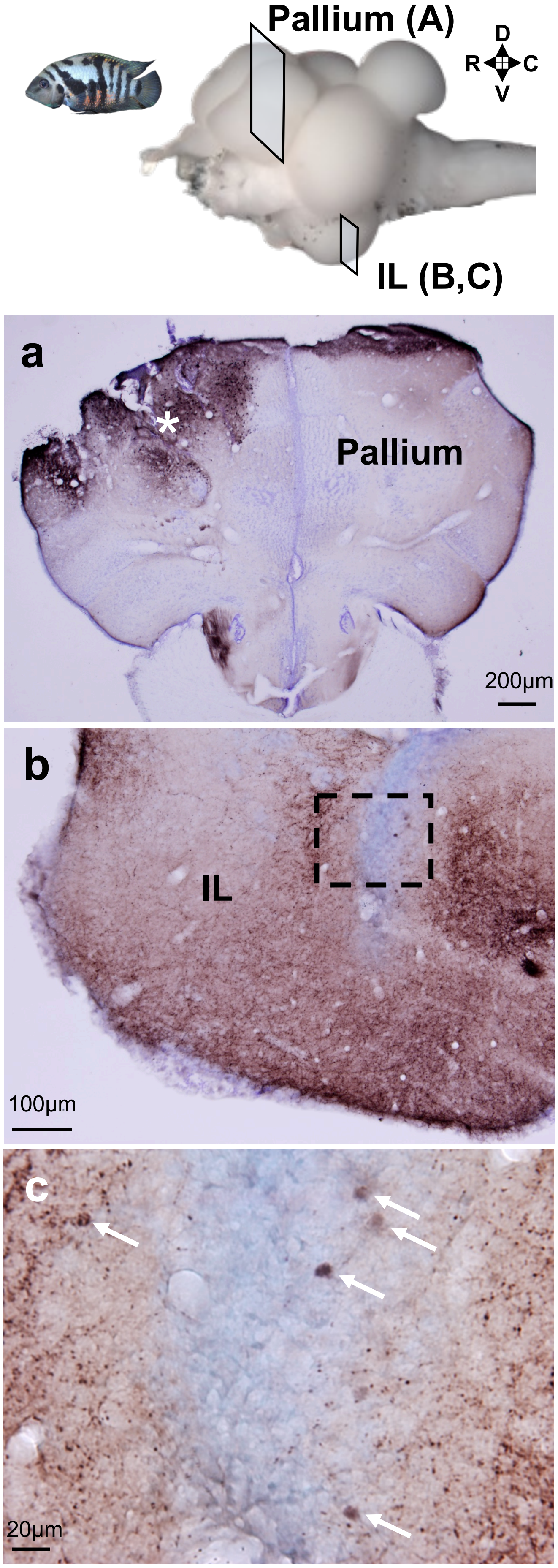

Supplementary Fig. 7. Connectivity of the pallium with the inferior lobe in the cichlid *A. nigrofasciata* visualized using biocytin.

Frontal sections showing biocytin tract-tracing in the cichlid *A. nigrofasciata.* The level of the sections is indicated at the top. Brown indicates biocytin, and purple indicates cresyl violet counterstaining.

(a) Level of the telencephalon, showing the injection site in the pallium (white asterisk).

(b) Ipsilateral inferior lobe (IL) showing abundant anterogradely labelled fiber terminals (dark brown). The dotted square delineates the area shown in (c).

(c) Higher magnification showing the retrogradely labelled cell bodies in the inferior lobe (white arrows).

Brain regions: IL: inferior lobe

R: rostral; C: caudal; D: dorsal; V: ventral.
