# Supplementary material for: Different ways of evolving tool-using brains in teleosts and amniotes
Source: Commun Biol. 2024 Jan 12;7:88. doi: 10.1038/s42003-023-05663-8 (PMC10786859; doi:10.1038/s42003-023-05663-8)
Supplement: Supplementary file 2 — Description of Additional Supplementary Files [file 42003_2023_5663_MOESM2_ESM.pdf]

Description of Additional Supplementary Files

File name: Supplementary Movie 1
Description: 3D selective visualization of inferior lobe fiber tracts in the wrasse (Choerodon anchorago) brain.

File name: Supplementary Movie 2
Description: 3D selective visualization of inferior lobe fiber tracts in the trout (Salmo trutta) brain.

File name: Supplementary Movie 3
Description: 3D selective visualization of inferior lobe fiber tracts in the Astyanax surface fish (Astyanax mexicanus) brain.

File name: Supplementary Movie 4
Description: 3D selective visualization of inferior lobe fiber tracts in the zebrafish (Danio rerio) brain.

File name: Supplementary Movie 5
Description: 3D selective visualization of inferior lobe fiber tracts in the cichlid (Neolamprologus brichardi) brain.

File name: Supplementary Data 1
Description: The source data behind the graphs in the paper.
