# Supplementary material for: Different ways of evolving tool-using brains in teleosts and amniotes
Source: Commun Biol. 2024 Jan 12;7:88. doi: 10.1038/s42003-023-05663-8 (PMC10786859; doi:10.1038/s42003-023-05663-8)
Supplement: Supplementary file 9 — Reporting Summary [file 42003_2023_5663_MOESM9_ESM.pdf]

nature portfolio

Corresponding author(s): Kei Yamamoto

Last updated by author(s): Oct 31, 2023

Reporting Summary

Nature Portfolio wishes to improve the reproducibility of the work that we publish. This form provides structure for consistency and transparency in reporting. For further information on Nature Portfolio policies, see our Editorial Policies and the Editorial Policy Checklist.

Statistics

For all statistical analyses, confirm that the following items are present in the figure legend, table legend, main text, or Methods section.

n/a	Confirmed	
☐	☒	The exact sample size (*n*) for each experimental group/condition, given as a discrete number and unit of measurement
☒	☐	A statement on whether measurements were taken from distinct samples or whether the same sample was measured repeatedly
☐	☒	The statistical test(s) used AND whether they are one- or two-sided<br>*Only common tests should be described solely by name; describe more complex techniques in the Methods section.*
☐	☒	A description of all covariates tested
☐	☒	A description of any assumptions or corrections, such as tests of normality and adjustment for multiple comparisons
☐	☒	A full description of the statistical parameters including central tendency (e.g. means) or other basic estimates (e.g. regression coefficient) AND variation (e.g. standard deviation) or associated estimates of uncertainty (e.g. confidence intervals)
☐	☒	For null hypothesis testing, the test statistic (e.g. *F*, *t*, *r*) with confidence intervals, effect sizes, degrees of freedom and *P* value noted<br>*Give P values as exact values whenever suitable.*
☒	☐	For Bayesian analysis, information on the choice of priors and Markov chain Monte Carlo settings
☒	☐	For hierarchical and complex designs, identification of the appropriate level for tests and full reporting of outcomes
☐	☒	Estimates of effect sizes (e.g. Cohen's *d*, Pearson's *r*), indicating how they were calculated

Our web collection on statistics for biologists contains articles on many of the points above.

Software and code

Policy information about availability of computer code

Data collection	No software was used
Data analysis	This study reports no original code.

For manuscripts utilizing custom algorithms or software that are central to the research but not yet described in published literature, software must be made available to editors and reviewers. We strongly encourage code deposition in a community repository (e.g. GitHub). See the Nature Portfolio guidelines for submitting code & software for further information.

Data

Policy information about availability of data

All manuscripts must include a data availability statement. This statement should provide the following information, where applicable:

- Accession codes, unique identifiers, or web links for publicly available datasets
- A description of any restrictions on data availability
- For clinical datasets or third party data, please ensure that the statement adheres to our policy

Source data is provided as Supplementary Data.

Human research participants

Policy information about studies involving human research participants and Sex and Gender in Research.

Reporting on sex and gender	NA
Population characteristics	NA
Recruitment	NA
Ethics oversight	NA

Note that full information on the approval of the study protocol must also be provided in the manuscript.

Field-specific reporting

Please select the one below that is the best fit for your research. If you are not sure, read the appropriate sections before making your selection.

☒ Life sciences ☐ Behavioural & social sciences ☐ Ecological, evolutionary & environmental sciences

For a reference copy of the document with all sections, see nature.com/documents/nr-reporting-summary-flat.pdf

Life sciences study design

All studies must disclose on these points even when the disclosure is negative.

Sample size	n=3 to n=5 brains of 11 fish species were used for isotropic fractionator experiments, based on availability of fish specimens and previously published sample sizes (see Olkowicz et al., Birds have primate like numbers of neurons in their forebrain), which demonstrated that such sample sizes yield applicable results.<br><br>For whole brain clearing, imaging and fiber tract 3D reconstruction , n=2 brains of each species studied were used, as this was a qualitative technique and since manual segmentation required to reconstruct fiber tracts in 3D was highly time-consuming.<br><br>Tract-tracing experiments with NeuroVue, Biocytin and BDA used n=2 to n=5 specimens, as availability of certain species was limited (in the case of NeuroVue), or because the experiments required brain surgery (in the case of biocytin and BDA injections), which require a rather large number of fish specimens to obtain one specimen with the right injection site. Survival rate for such surgeries is rather low, which also explains the low sample size. This is a qualitative rather than a quantitative method, which also justifies this sample size.
Data exclusions	No data were excluded from the analysis.
Replication	The data collected in this study was not replicated.
Randomization	There was no need for randomization in this study.
Blinding	Blinding the species of fish for the isotropic fractionator was not possible, as the experimenter counting the cells was the one who performed the brain sampling and dissection.<br><br>Fiber tracts for the 3D reconstruction of brain fibers were segmented and visualized by a blind experimenter to avoid over-interpretation of the data.

Reporting for specific materials, systems and methods

We require information from authors about some types of materials, experimental systems and methods used in many studies. Here, indicate whether each material, system or method listed is relevant to your study. If you are not sure if a list item applies to your research, read the appropriate section before selecting a response.

Materials & experimental systems

n/a	Involved in the study
☐	☒ Antibodies
☒	☐ Eukaryotic cell lines
☒	☐ Palaeontology and archaeology
☐	☒ Animals and other organisms
☒	☐ Clinical data
☒	☐ Dual use research of concern

Methods

n/a	Involved in the study
☒	☐ ChIP-seq
☒	☐ Flow cytometry
☒	☐ MRI-based neuroimaging

Antibodies

Antibodies used

Anti-NeuN (rabbit polyclonal), Merck Cat. No. ABN78; Anti-NeuN (rabbit polyclonal) Antibody, Cy3 Conjugate Merck Cat. No. ABN78C3; Anti-NeuN (rabbit monoclonal) antibody, abcam Cat. No. ab177487; Anti-NeuN (mouse monoclonal) Antibody, Chemicon Cat. No. MAB377

Validation

-ABN78: "RNA binding protein fox-1 homolog 3 (UniProt Q8BIF2; also known as Fox-1 homolog C, Fox-3, Hexaribonucleotide-binding protein 3, NeuN, Neuronal nuclei) is encoded by the Rbfox3 (also known as D11Bwg0517e, Hrnbp3) gene (Gene ID 52897) in murine species. The RNA-binding Fox (Rbfox) family of splicing factors is comprised of three members, Rbfox1 (Fox-1 or A2BP1), Rbfox2 (Fox-2 or RBM9), and Rbfox3 (Fox-3, HRNBP3 or NeuN). Rbfox proteins regulate splicing of many neuronal transcripts (pre-mRNAs) by binding the sequence (U)GCAUG in introns flanking alternative exons. A (U)GCAUG motif that lies downstream of the alternative exon generally promotes Rbfox-dependent exon inclusion, whereas an upstream motif will usually repress exon inclusion. Originally characterized as a marker of post-mitotic neurons and named neuronal nuclei (NeuN), Rbfox3 plays a role in promoting neuronal differentiation through alternative splicing of Numb pre-mRNA during brain development. Rbfox3-knockout in mice is reported to result in defective hippocampal gene expression, defects in synaptic transmission and plasticity in the dentate gyrus, as well as increased seizure susceptibility and decreased anxiety-related behaviors. Likewise, human RBFOX3 gene mutations have been linked to neurodevelopmental delay, cognitive impairments, autistic features, and epilepsy." from the manufacturer website: https://www.merckmillipore.com/product/Anti-NeuN-Antibody-rabbit,MM_NF-ABN78

-ABN78C3: "ABN78 is a rabbit polyclonal version of the Anti-NeuN, clone A60 (MAB377), NeuN is a highly characterized and cited mouse monoclonal antibody that specifically recognizes the DNA-binding, neuron-specific protein NeuN, which is present in most CNS and PNS neuronal cell types of all vertebrates tested. NeuN protein distributions are apparently restricted to neuronal nuclei, perikarya and some proximal neuronal processes in both fetal and adult brain although, some neurons fail to be recognized by NeuN at all ages: INL retinal cells, Cajal-Retzius cells, Purkinje cells, inferior olivary and dentate nucleus neurons, and sympathetic ganglion cells are examples. Immunohistochemically detectable NeuN protein first appears at developmental timepoints that correspond with the withdrawal of the neuron from the cell cycle and/or with the initiation of terminal differentiation of the neuron. Immunoreactivity appears around E9.5 in the mouse neural tube and is extensive throughout the developing nervous system by E12.5. Strong nuclear staining suggests a nuclear regulatory protein function; however, no evidence currently exists as to whether the NeuN protein antigen has a function in the distal cytoplasm or whether it is merely synthesized there before being transported back into the nucleus. No difference between protein isolated from purified nuclei and whole brain extract on immunoblots has been found." from the manufacturer website: https://www.merckmillipore.com/product/Anti-NeuN-rabbit-Antibody-Cy3-Conjugate,MM_NF-ABN78C3

-ab177487: "Produced recombinantly (animal-free) for high batch-to-batch consistency and long term security of supply. Suitable for: Flow Cyt (Intra), IHC (PFA fixed), mIHC, IHC-P, WB, ICC/IF, IHC-Fr. Reacts with: Mouse, Rat, Sheep, Goat, Cat, Dog, Human, Zebrafish, Common marmoset. Positive control: WB: Mouse brain, mouse cerebellum, rat cerebellum and human fetal brain tissue lysates. ICC/IF: SH-SY-5Y and Mouse primary neuron cells. IHC-P: Human cerebellum, human gliocytoma tissue. mIHC: Human cerebellum tissue IHC-Fr: Mouse dentate gyrus tissue. Flow Cyt (intra): U-87 MG cells." from manufacturer website: https://www.abcam.com/products/primary-antibodies/neun-antibody-epr12763-neuronal-marker-ab177487.html

-MAB377: "NeuN antibody (NEUronal Nuclei; clone A60) specifically recognizes the DNA-binding, neuron-specific protein NeuN, which is present in most CNS and PNS neuronal cell types of all vertebrates tested. NeuN protein distributions are apparently restricted to neuronal nuclei, perikarya and some proximal neuronal processes in both fetal and adult brain although, some neurons fail to be recognized by NeuN at all ages: INL retinal cells, Cajal-Retzius cells, Purkinje cells, inferior olivary and dentate nucleus neurons, and sympathetic ganglion cells are examples (Mullen et al., 1992; Wolf et al., 1996). Immunohistochemically detectable NeuN protein first appears at developmental timepoints that correspond with the withdrawal of the neuron from the cell cycle and/or with the initiation of terminal differentiation of the neuron (Mullen et al., 1992). Immunoreactivity appears around E9.5 in the mouse neural tube and is extensive throughout the developing nervous system by E12.5. Strong nuclear staining suggests a nuclear regulatory protein function; however, no evidence currently exists as to whether the NeuN protein antigen has a function in the distal cytoplasm or whether it is merely synthesized there before being transported back into the nucleus. No difference between protein isolated from purified nuclei and whole brain extract on immunoblots has been found (Mullen et al., 1992)." from manufacturer website: https://www.sigmaaldrich.com/product/mm/mab377

Animals and other research organisms

Policy information about studies involving animals; ARRIVE guidelines recommended for reporting animal research, and Sex and Gender in Research

Laboratory animals

8 species of teleost were used: the zebrafish Danio rerio (n=9), the medaka Oryzias latipes (n=5), the Astyanax surface fish Astyanax

Laboratory animals	mexicanus (n=10), the trout Salmo trutta (n=6), and the cichlids Maylandia zebra (n=3), Neolamprologus brichardi (n=18), Ophthalmotilapia boops (n=3), and Amatitlania nigrofasciata (n=10).
Wild animals	3 wrasse species (Choerodon anchorago, n=9, Labroides dimidiatus, n=3, Thalassoma hardwicke, n=3) were obtained from a commercial provider, and were wild caught in Indonesia before being shipped to France, where they were euthanized in order to collect brain samples.
Reporting on sex	*Indicate if findings apply to only one sex; describe whether sex was considered in study design, methods used for assigning sex. Provide data disaggregated for sex where this information has been collected in the source data as appropriate; provide overall numbers in this Reporting Summary. Please state if this information has not been collected. Report sex-based analyses where performed, justify reasons for lack of sex-based analysis.*
Field-collected samples	This study did not involve samples collected from the field.
Ethics oversight	All procedures were conducted in compliance with the official regulatory standards of the French Government and in compliance with the official Japanese regulations for research on animal, and the regulations on Animal Experiments in Nagoya University.

Note that full information on the approval of the study protocol must also be provided in the manuscript.
